# Flexible solar cells based on foldable silicon wafers with blunted edges

**DOI:** 10.1038/s41586-023-05921-z

**Published:** 2023-05-24

**Authors:** Wenzhu Liu, Yujing Liu, Ziqiang Yang, Changqing Xu, Xiaodong Li, Shenglei Huang, Jianhua Shi, Junling Du, Anjun Han, Yuhao Yang, Guoning Xu, Jian Yu, Jiajia Ling, Jun Peng, Liping Yu, Bin Ding, Yuan Gao, Kai Jiang, Zhenfei Li, Yanchu Yang, Zhaojie Li, Shihu Lan, Haoxin Fu, Bin Fan, Yanyan Fu, Wei He, Fengrong Li, Xin Song, Yinuo Zhou, Qiang Shi, Guangyuan Wang, Lan Guo, Jingxuan Kang, Xinbo Yang, Dongdong Li, Zhechao Wang, Jie Li, Sigurdur Thoroddsen, Rong Cai, Fuhai Wei, Guoqiang Xing, Yi Xie, Xiaochun Liu, Liping Zhang, Fanying Meng, Zengfeng Di, Zhengxin Liu

**Affiliations:** 1 Research Center for New Energy Technology, Shanghai Institute of Microsystem and Information Technology, Chinese Academy of Sciences, Shanghai, China; 2 University of Chinese Academy of Sciences, Beijing, China; 3 Institute of Metals, College of Material Science and Engineering, Changsha University of Science and Technology, Changsha, China; 4 Division of Physical Science and Engineering, King Abdullah University of Science and Technology, Thuwal, Saudi Arabia; 5 Division of Computer, Electrical and Mathematical Science and Engineering, King Abdullah University of Science and Technology, Thuwal, Saudi Arabia; 6 School of Physical Science and Technology, ShanghaiTech University, Shanghai, China; 7 Tongwei Solar Company, Chengdu, China; 8 Aerospace Information Research Institute, Chinese Academy of Sciences, Beijing, China; 9 Institute of Photovoltaics, Southwest Petroleum University, Chengdu, China; 10 UISEE Technologies, Shanghai, China; 11 Jiangsu Key Laboratory of Carbon-Based Functional Materials and Devices, Institute of Functional Nano and Soft Materials, Soochow University, Suzhou, China; 12 Institute of Solid Mechanics, Beihang University, Beijing, China; 13 State Key Laboratory of Transducer Technology, Shanghai Institute of Microsystem and Information Technology, Chinese Academy of Sciences, Shanghai, China; 14 Key Laboratory of Wireless Sensor Networks and Communications of CAS, Shanghai Institute of Microsystem and Information Technology, Chinese Academy of Sciences, Shanghai, China; 15 School of Materials Science and Engineering, Jiangsu Collaborative Innovation Center of Photovoltaic Science and Engineering, Changzhou University, Changzhou, China; 16 Paul-Drude-Institut für Festkörperelektronik, Leibniz Institut, Berlin, Germany; 17 College of Energy, Soochow Institute for Energy and Materials Innovations, Soochow University, Suzhou, China; 18 The Interdisciplinary Research Center, Shanghai Advanced Research Institute, Chinese Academy of Sciences, Shanghai, China; 19 Polar Research Institute of China, Shanghai, China; 20 State Key Laboratory of Functional Materials for Informatics, Shanghai Institute of Microsystem and Information Technology, Chinese Academy of Sciences, Shanghai, China

**Keywords:** Solar cells, Solar cells

## Abstract

Flexible solar cells have a lot of market potential for application in photovoltaics integrated into buildings and wearable electronics because they are lightweight, shockproof and self-powered. Silicon solar cells have been successfully used in large power plants. However, despite the efforts made for more than 50 years, there has been no notable progress in the development of flexible silicon solar cells because of their rigidity^1–4^. Here we provide a strategy for fabricating large-scale, foldable silicon wafers and manufacturing flexible solar cells. A textured crystalline silicon wafer always starts to crack at the sharp channels between surface pyramids in the marginal region of the wafer. This fact enabled us to improve the flexibility of silicon wafers by blunting the pyramidal structure in the marginal regions. This edge-blunting technique enables commercial production of large-scale (>240 cm^2^), high-efficiency (>24%) silicon solar cells that can be rolled similarly to a sheet of paper. The cells retain 100% of their power conversion efficiency after 1,000 side-to-side bending cycles. After being assembled into large (>10,000 cm^2^) flexible modules, these cells retain 99.62% of their power after thermal cycling between −70 °C and 85 °C for 120 h. Furthermore, they retain 96.03% of their power after 20 min of exposure to air flow when attached to a soft gasbag, which models wind blowing during a violent storm.

## Abstract (web summary)

Modules of foldable crystalline silicon solar cells retain their power-conversion efficiency after being subjected to bending stress or exposure to air-flow simulations of a violent storm.

## Main

Silicon is the most abundant semiconducting element in Earth’s crust; it is made into wafers to manufacture approximately 95% of the solar cells in the current photovoltaic market^5^. However, these cells are brittle and crack under bending stress, which limits their large-scale use for flexible applications. At present, thin-film solar cells made from amorphous silicon, Cu(In,Ga)Se_2_, CdTe, organics and perovskites exhibit flexibility^6–9^ but their use is limited because of their low power conversion efficiency (PCE), release of toxic materials into the environment, inferior performance in the case of large areas and unstable operating conditions. Therefore, many available flexible solar cells have not attracted customers and most companies that manufactured them have gone out of business. In this study, we propose a morphology engineering method to fabricate foldable crystalline silicon (c-Si) wafers for large-scale commercial production of solar cells with remarkable efficiency.

## Fabrication of foldable c-Si wafers

**Extended Data Fig. 1:**
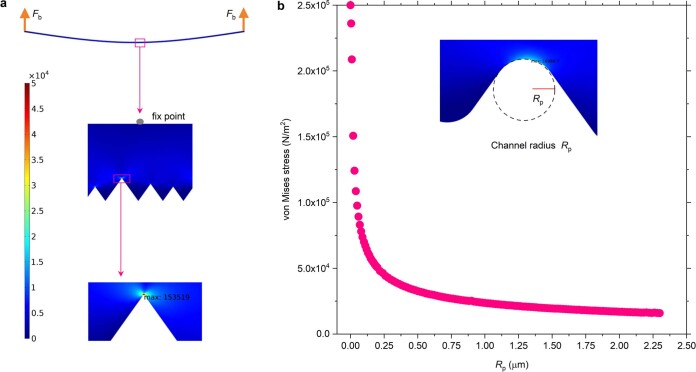
Deformation and stress calculated by the solid mechanics module in COMSOL Multiphysics. **a**, 2D simulation of a textured c-Si wafer whose length and thickness were set as 1 cm and 60 μm, respectively. Three points near the middle of the upper surface were fixed to exclude rigid-body displacement. Vertical forces *F*_b_ = 1.2 mN were loaded at the endpoints of the wafer. The bright colour indicates that most of the von Mises stress was concentrated in the sharp channels between pyramids. **b**, The maximum von Mises stress was simulated as a function of the channel radius *R*_p_, which was tuned by a blunting treatment in an acid solution.

**Extended Data Fig. 2:**
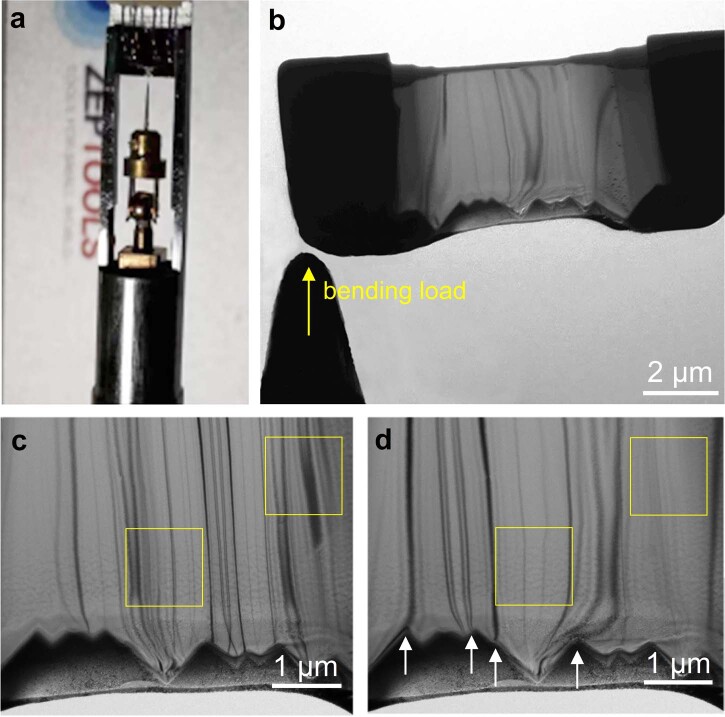
*In situ* bending test by TEM. **a**, A stress holder for the *in situ* bending test during TEM. **b**, A tungsten tip controlled by the piezomanipulator was used to handle the left side of the FIB c-Si foil. **c**, Before loading the bending force, the bend contours were randomly distributed on the c-Si foil. **d**, After loading the bending force, these bend contours collected in the sharp channels between pyramids (white arrows), indicating that most stress concentrated in these sharp channels. The yellow squares implied that most stress shifted to nearby sharp channels after the bending force was loaded. Here, the dark fringes resulted from stress-induced lattice deformations.

Our first goal was to fabricate foldable c-Si wafers with a strong light-harvesting ability. Reducing the thickness of a wafer can improve its flexibility^10^, but there is a trade-off between thickness and light-harvesting efficiency because c-Si is a semiconductor with an indirect optical bandgap. By using saw-damage removal^11^, we reduced the thickness of a 160-μm wafer to 60 μm. Although the wafer began to exhibit flexibility similar to that of a sheet of paper (Supplementary Fig. 1), it was not suitable for solar cell fabrication because more than 30% of the incident sunlight was reflected by its glossy surface^12^. Chemically texturing microscale pyramids on c-Si surfaces has been widely used as an efficient strategy to reduce the reflectivity to less than 10% owing to Lambertian light trapping^13^. However, when bending forces were applied to such textured wafers, the maximum stress was located in the sharp channels between the pyramids, as observed in the simulation with the solid mechanics module in COMSOL Multiphysics (Extended Data Fig. 1a). This result was consistent with an in situ image obtained using transmission electron microscopy (TEM), in which the bending stress accumulated in the channels between pyramids under a typical bending load exerted by a micromanipulator (Extended Data Fig. 2). Further simulations revealed that a slight increase in the channel radius (*R*_p_) from 0 μm to 2.3 μm led to a rapid reduction in the maximum stress from 0.25 MPa to 0.016 MPa (Extended Data Fig. 1b). But this blunting treatment increased the reflectivity to more than 30% (Supplementary Fig. 2), which was unfavourable for light harvesting. This was confirmed by optical simulations of the devices, in which the blunted wafers showed inferior antireflection and light trapping (Supplementary Fig. 3).

**Extended Data Fig. 3:**
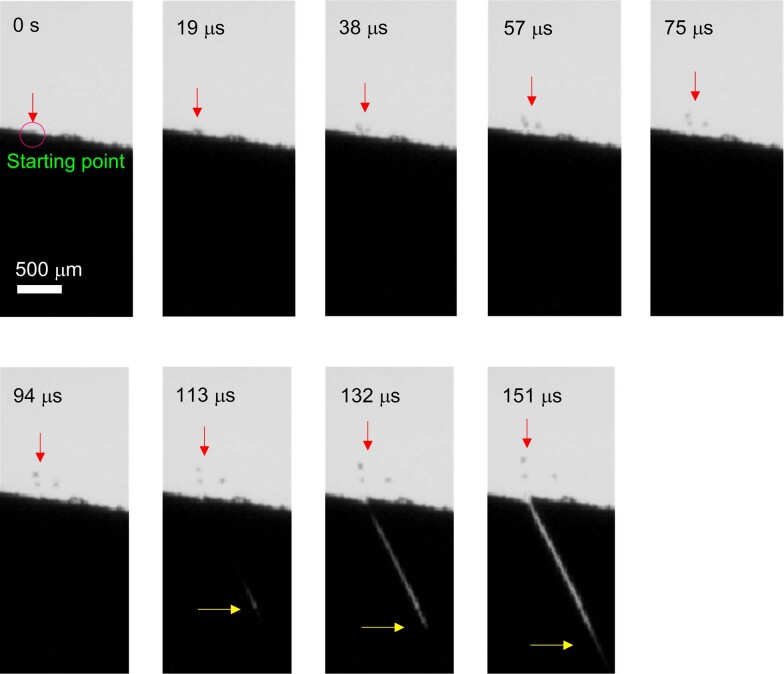
Cracking process recorded by an ultrahigh-speed video camera. Photographs of cracking of a 60 μm textured c-Si wafer on a time scale of 0−151 μs. The vertical arrows point to three emitted silicon particles, while the lateral arrows point to the endpoint of an evolving fracture. The circle highlights the site of initiation of the cracking.

Next, we used an ultrahigh-speed video camera to investigate the cracking process of a wafer. As shown in Extended Data Fig. 3, the camera recorded a long fracture in the snapshots taken at 113, 132 and 151 μs (yellow arrows). If we assume that the cracking initiates from a point on the edge (circle), then the propagation velocities of the crack can be estimated from the evolution of the fracture length as 33.2, 33.6 and 33.0 m s^−1^, respectively. The consistency of these values supported our hypothesis. Furthermore, the camera recorded three silicon particles being ejected from the edge of the wafer (red arrows); their initial positions coincided with the point at which the cracking initiated. This extra evidence confirmed that the cracking initiated at the edge of the wafer, which explains why most linear cracks in electroluminescence images begin from the edges of silicon solar cells^14^ (Supplementary Fig. 4).

**Fig. 1:**
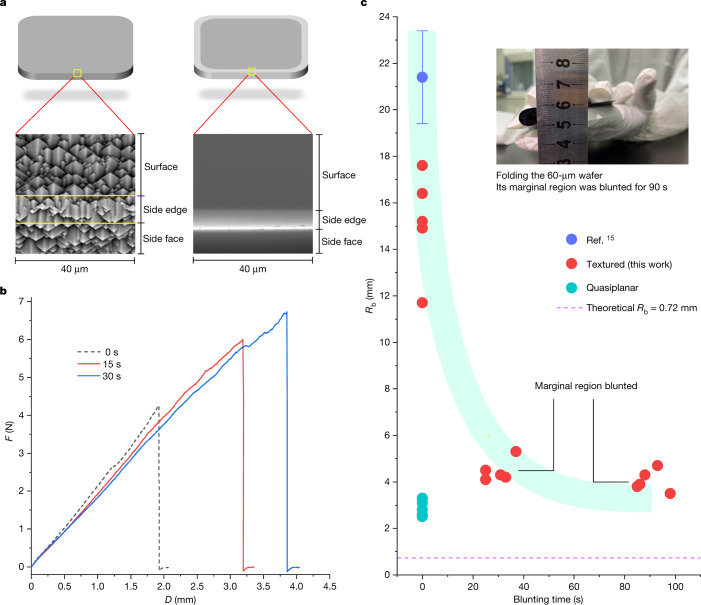
Foldable wafers. **a**, SEM images of a textured c-Si wafer. The sharp pyramids in the marginal region were efficiently removed by an acid solution. **b**, Load–vertical displacement (*F*–*D*) curves of 140-μm textured c-Si wafers, in which the marginal regions were blunted in 10 vol% HF:90 vol% HNO_3_ solution for 0, 15 and 30 s. **c**, Textured c-Si wafers (60 μm) with pyramids on the surface. Their bending radius (*R*_b_) at the cracking moment is plotted as a function of the blunting time in 10 vol% HF:90 vol% HNO_3_ solution. For comparison, the *R*_b_ values of 60-μm textured^15^ and quasiplanar c-Si wafers are shown. We also calculated the theoretical *R*_b_ of a 60-μm c-Si wafer as *R*_b_ = *Ed*/2*σ*, where *E*, *d* and *σ* are the modulus of elasticity, wafer thickness and tensile yield strength, respectively. Inset, 60-μm textured wafer of size 15.6 cm × 15.6 cm, in which the marginal region was blunted for 90 s in the acid solution.

On the basis of the cracking characteristics discussed above, we considered blunting the sharp channels in the marginal region instead of the whole wafer to improve the flexibility of the silicon wafers (Fig. 1a). The three-point bending test results in Fig. 1b show that the 15-s and 30-s edge-blunting treatments increased the vertical displacement of the wafer at the cracking moment from 1.92 mm to 3.20 mm and 3.86 mm, respectively. Consistent with these results, after the approximately 2-mm-wide marginal region of a 60-μm textured wafer was blunted for 0, 30 and 90 s in 10 vol% HF:90 vol% HNO_3_ solution, the critical bending radius (*R*_b_) at the cracking moment considerably decreased from 15.2 ± 2 mm (this study) or 21.4 ± 2 mm (ref. ^15^) to approximately 4.0 mm, which approached the theoretical limit of 0.72 mm (Fig. 1c). As expected, we could fold the wafer around its centre with *R*_b_ = 4.0 mm (Fig. 1c, inset) and vigorously shake it like a sheet of flexible paper (Supplementary Video 1). The improvement in flexibility was also supported by atomistic simulations: cracking of the untreated wafer initiated under a loading strain of 9.3%, but this value rapidly increased to 17.3% (Supplementary Videos 2–5) even when the sharp channels were blunted to a very small *R*_p_ of 15.81 nm.

## Flexibility mechanism

**Fig. 2:**
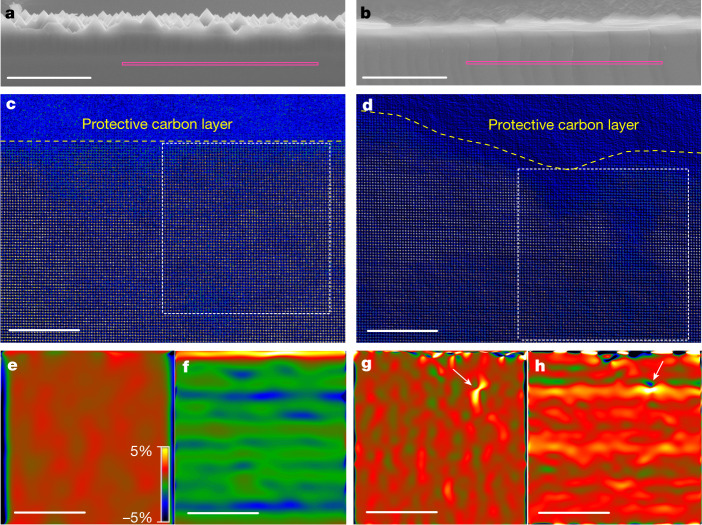
GPA of the fracture surfaces. **a**,**b**, Morphology of the fracture surface of a wafer with sharp (**a**) and round (**b**) pyramids. The pink lines mark the locations at which the top surface of the fracture was protected and lifted out for TEM observations using an FIB. **c,d**, High-resolution STEM-HAADF images showing the atomic arrangement at a depth of dozens of atoms viewed along the [001] direction from the fracture surface of the wafer with sharp (**c**) and round (**d**) pyramids, in which a protective carbon layer was deposited on the fracture surface. The GPA regions are highlighted by dashed squares. **e**,**f**, Elastic lattice strain distribution in the *x* direction (**e**) and *y* direction (**f**) of the wafer with sharp pyramids. **g**,**h**, Elastic lattice strain distribution in the *x* direction (**g**) and *y* direction (**h**) of the wafer with round pyramids. White arrows mark the great dilatation strain. Positive and negative values represent lattice expansion and contraction, respectively. The *x* direction is parallel and the *y* direction is perpendicular to the fracture surfaces marked in **a** and **b**, respectively. Scale bars, 5 μm (**a**,**b**); 5 nm (**c**–**h**).

**Extended Data Fig. 4:**
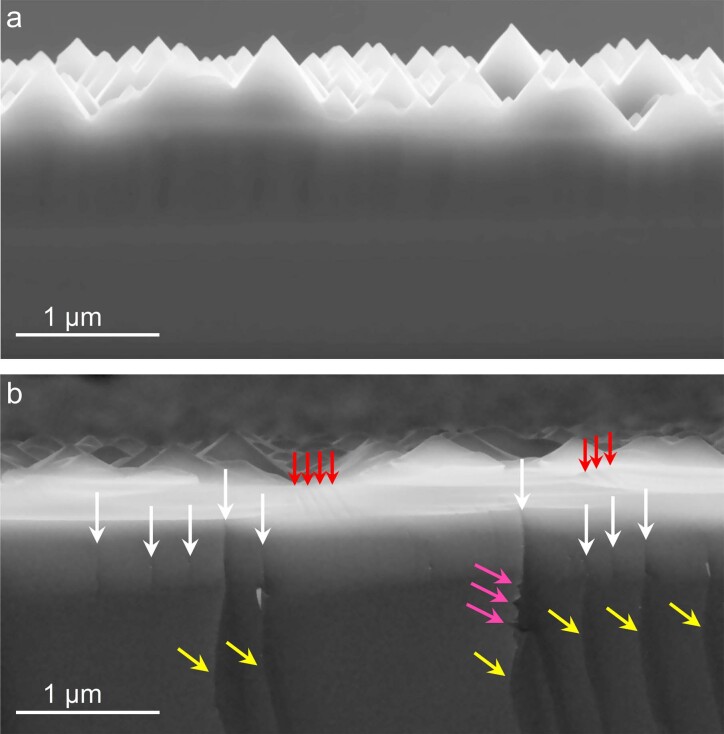
Morphology of fracture surfaces. **a**, Fracture surface of a 60 μm c-Si wafer with sharp channels between pyramids. **b**, Fracture surface of a 60 μm c-Si wafer whose channels between pyramids were blunted in 10 vol% HF/90 vol% HNO_3_ solution for 30 s. The multiple cleavage sites in the fracture consumed more energy before the initiation of cracking.

**Extended Data Fig. 5:**
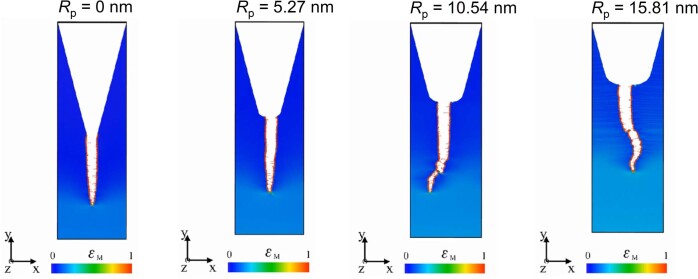
Simulated fracture characteristics. Together with the increase of *R*_p_ from 0 to 15.81 nm, the fractured path dually becomes more tortuous at a strain of 27.5%. Some jagged notches are also observed, in consistent with experimental results in Extended Data Fig. 4b.

To understand the flexibility of the c-Si wafers shown in Fig. 1c, we broke two wafers by applying bending forces to find out the morphologies of the fracture surfaces. The image obtained by scanning electron microscopy (SEM) of the untreated wafer in Fig. 2a shows a flat cleavage surface, whereas the SEM image of the blunted wafer in Fig. 2b shows a fracture surface with multiple cleavage sites and a high density of microcracks, which is also evident in the stepwise focused ion beam (FIB) image of the fracture surface (Supplementary Fig. 5). In the magnified view of the fracture surface with multiple cleavage sites (Extended Data Fig. 4), we observed large cracks propagating along complicated paths in deeper regions of the blunted wafer (yellow arrows) and some jagged notches (pink arrows), which were in good agreement with the atomistic simulations (Extended Data Fig. 5). Within a depth of around 500 nm below the top surface (white arrows), secondary shear banding lines (red arrows) were generated in a direction different from that of the dominant cracks (yellow arrows; Extended Data Fig. 4). These features indicated the development of a complex stress state during the cracking process, which was similar to the restoration of deformability of brittle metallic glass realized by triggering secondary shear banding^16^. Physically, these bumpy cleavage processes consumed more energy before the initiation of cracking; thus, they accounted for the robust behaviour that provided protection against the violent folding (Fig. 1c).

Using spherical aberration-corrected TEM, we analysed the lattice strains beneath the fracture surface. After depositing a protective carbon layer on the fresh fracture surface, we obtained atomic-resolution images using high-angle annular dark field scanning transmission electron microscopy (HAADF-STEM) of the untreated (Fig. 2c) and blunted (Fig. 2d) wafers. The rough fracture surface marked by a yellow dashed line in Fig. 2d indicates that the blunted wafer underwent more elastic and plastic strain during the cracking process. Because some of the lattice strain caused by lattice distortion could be preserved in dozens of atomic layers beneath the fracture surface, we could analyse the residual strain as an indicator of the cracking mode. Figure 2e,f shows the geometric phase analyses^17^ (GPAs) of the area beneath the fracture surface of the untreated wafer (Fig. 2c). This wafer showed general tensile strain in the *x* direction and compressive strain in the *y* direction but showed remarkable dilatation strain in the *y* direction within a few atomic layers of the top surface. These characteristics pertained to a typical brittle fracture mode. By contrast, Fig. 2g,h shows that the fracture surface of the blunted wafer had larger lattice strain variations in both the *x* and *y* directions; the great dilatation strain is marked by white arrows. This feature suggests that complex cracking can result in much larger lattice expansion. These findings prove that the fracture behaviour of c-Si wafers can be manipulated by tuning the sharpness of the channels between surface pyramids, which modifies the stress state and deformation mechanism under bending loads. As a consequence, in this study, the blunting treatment greatly mitigated the intrinsic brittleness of the c-Si wafer, which led to a transition of the fracture mechanism from intrinsic brittle cleavage fracture to shear banding with steps and cracks.

## Solar cell (module) characterization

Next, we fabricated the foldable c-Si wafers into solar cells. The most widely used industrial silicon solar cells include passivated emitter and rear cells^18^, tunnelling oxide passivated contact^19^ solar cells and amorphous–crystalline silicon heterojunction^20^ (SHJ) solar cells. As shown in Supplementary Fig. 6, unlike passivated emitter and rear cells and tunnelling oxide passivated contact solar cells, which have asymmetric structure designs and are fired at a high temperature of 800 ± 20 °C, SHJ solar cells have a symmetric structural designs and are fired at a low temperature of 180 ± 5 °C. Therefore, SHJ technology is more suitable for manufacturing flexible solar cells because it is free from edge warping caused by inner stress during the firing process.

**Fig. 3:**
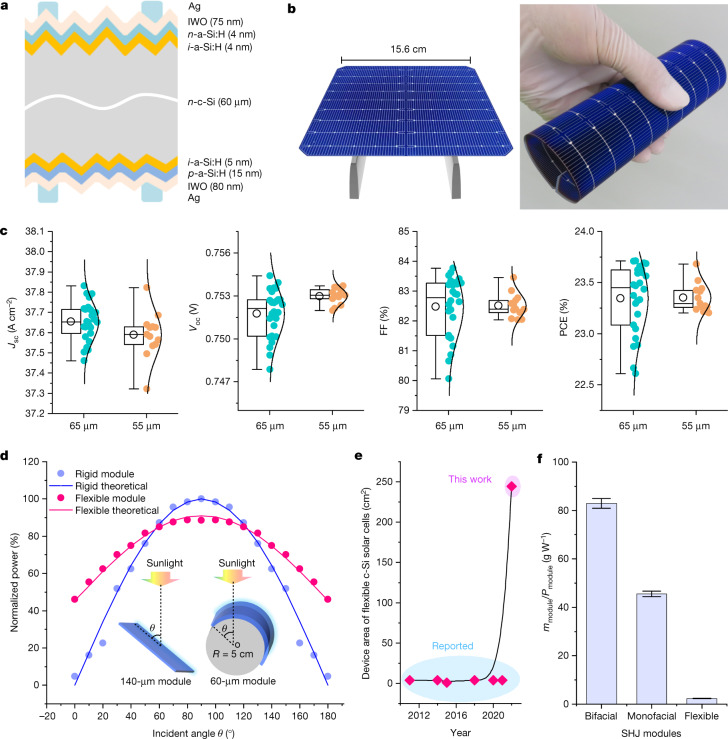
Solar cell (module) performance. **a**, Schematic of the architecture of the SHJ solar cells used in this study. IWO, tungsten-doped indium oxide. **b**, Photographs of a 15.6 cm × 15.6 cm flexible SHJ solar cell. **c**, *J*_sc_, *V*_oc_, FF and PCE of 65-μm and 55-μm SHJ solar cells. The top lines, bottom lines, lines in the box, circles and boxes represent maximum values, minimum values, median values, mean values and 25–75% distributions, respectively. **d**, Normalized power of two mini-modules tested as a function of the incident angle of light *θ*: a rigid module assembled from a 140-μm SHJ cell and a flexible module assembled from a 60-μm SHJ cell. The latter was attached to a black cylinder with a radius of 5 cm. The theoretical power of the rigid module was given by *P*(*θ*) = sin *θ*, whereas that of the flexible module was given by *P*(*θ*) = 0.455 × [1 + sin *θ*]. We collected experimental data from 0° to 90°; other data from 90° to 180° were symmetrically obtained by applying *P*(*θ*) = *P*(180° − *θ*). **e**, Evolution of the device area of flexible c-Si solar cells. **f**, Mass-to-power ratio of bifacial, monofacial and flexible SHJ modules tested under standard conditions, where *m*_module_ and *P*_module_ are the mass and power of the modules.

**Extended Data Fig. 6:**
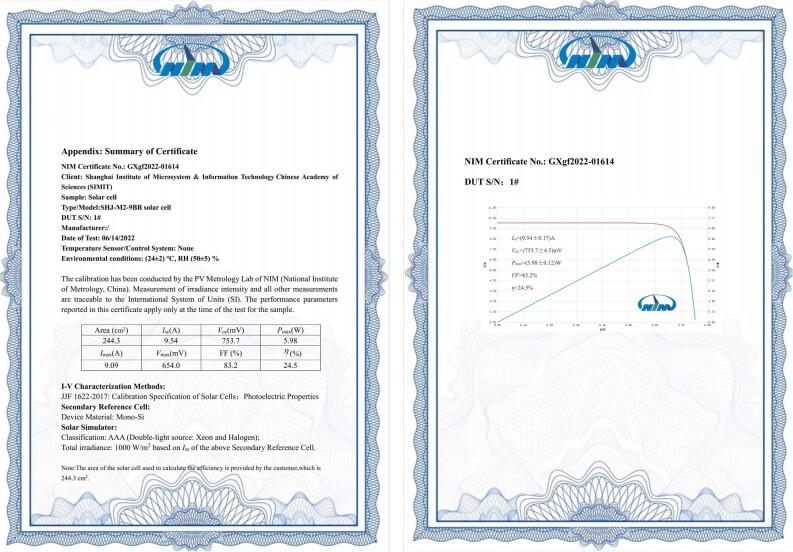
Certificate report of a flexible SHJ solar cell. Certificate of test results for a 60 μm flexible SHJ solar cell, whose short-circuit current density was 39.05 mA·cm^−2^ under AM1.5 illumination.

**Extended Data Fig. 7:**
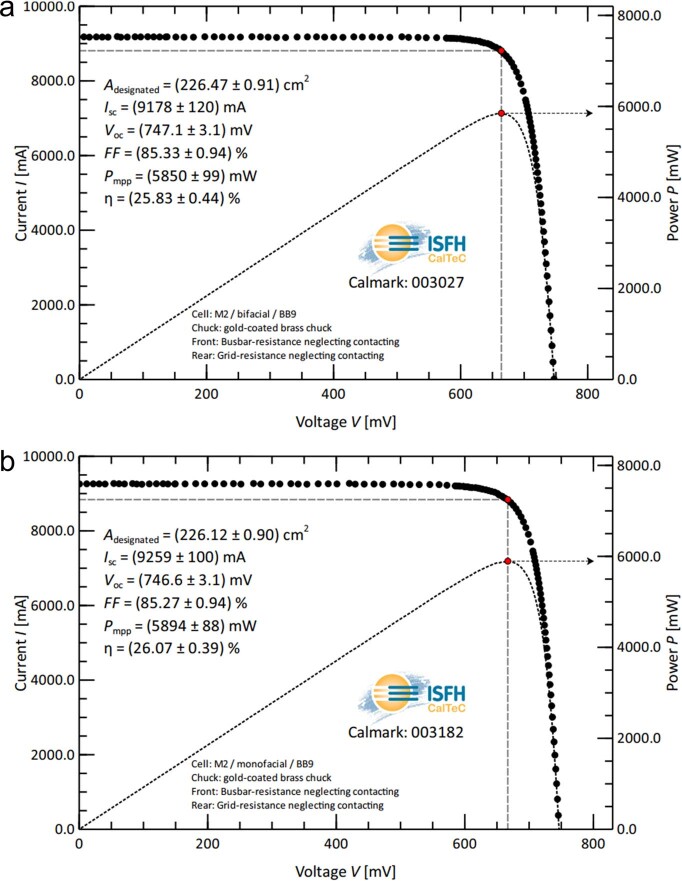
Certificate report of brittle SHJ solar cells. **a**, Certificate of test results for a 140 μm brittle SHJ solar cell, whose short-circuit current density was 40.53 mA·cm^−2^ under AM1.5 illumination. **b**, Certificate of test results for a 140 μm brittle SHJ solar cell with Ag reflector on the rear side, whose short-circuit current density was 40.95 mA·cm^−2^ under AM1.5 illumination.

Figure 3a shows the architecture of fabricated SHJ solar cells; their edges allow them to be rolled to more than 360° (Fig. 3b). The photovoltaic performance of the 65-μm and 55-μm devices is shown in Fig. 3c. The short-circuit current density (*J*_sc_), open-circuit voltage (*V*_oc_), fill factor (FF) and PCE are 37.65 ± 0.09 mA cm^−2^, 0.752 ± 0.002 V, 82.40 ± 0.99% and 23.31 ± 0.33%, respectively, for the 65-μm device. The corresponding *J*_sc_, *V*_oc_, FF and PCE values for the 55-μm device are 37.59 ± 0.11 mA cm^−2^, 0.753 ± 0.001 V, 82.51 ± 0.39% and 23.35 ± 0.13%, respectively. These PCEs are higher than the value of 19.67 ± 0.34% for flexible SHJ solar cells fabricated using 60-μm quasiplanar wafers because of the higher *J*_sc_ values of 65-μm (37.65 mA cm^−2^) and 55-μm (37.59 mA cm^−2^) devices compared with that of the 65-μm wafer (31.45 mA cm^−2^; Supplementary Fig. 7). After capping a 110-nm MgF_2_ antireflective layer on the side exposed to sunlight, we submitted one flexible cell to an independent test centre and obtained a certified PCE of 24.50% for a 244.3-cm^2^ wafer (Extended Data Fig. 6). Although this value was lower than that (25.83%) of a thick cell (Extended Data Fig. 7) because it was affected by the inferior light-harvesting ability of the thinner wafer^21^, it was a remarkable PCE compared with that of the current flexible solar cells fabricated from other cost-effective materials. However, considering the *V*_oc_ of 750 mV for a 98-μm wafer and implied *V*_oc_ of about 760 mV for 40-μm wafers^22,23^, the PCE in this work should be further improved through better surface passivation.

Two mini modules were assembled to compare their performance: a rigid module encapsulating a 140-μm SHJ cell and a flexible module encapsulating a 60-μm SHJ cell. The latter was attached to a black cylinder with a radius of 5 cm. Their power was measured as a function of the incident angle of light (Fig. 3d). Although the flexible module showed a lower power at normal incidence (90°), its integrated electricity generation from 0° to 180° was 17% greater than that of the rigid module. Given that the 140-μm wafer accounted for approximately 50% of the device cost, the use of a 60-μm wafer reduced the production cost by approximately 29%. Overall, the flexible technology developed in this study reduced the levelized cost of energy by approximately 39% (23%) at the solar cell (module) level. Moreover, recent years have seen a rapid decrease in the diameter of diamond wire saws from about 80 μm to 40 μm, which can successfully cut 115 ± 5 μm wafers with a high product yield. The ability to produce thinner wafers with less kerf loss should contribute to reducing CO_2_ emissions.

**Extended Data Fig. 8:**
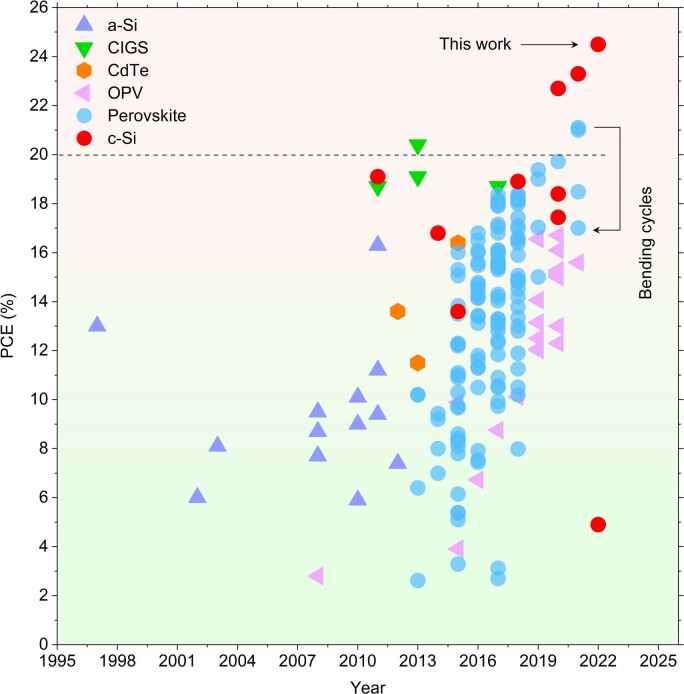
PCE of flexible solar cells. Emerging PCEs of flexible solar cells in the literature. Bending cycles decreased the PCE of the perovskite cell from 21% to 17%. For comparison, the certified PCE in this study of a 244.3 cm^2^ c-Si wafer is also displayed. The dashed line indicates an efficiency boundary of 20%.

Extended Data Fig. 8 shows the rapid development of flexible solar cells during the past two decades^2,15,24–38^. Our device highlights an advancement in the research field of flexible cells because most reported PCEs are below 20%. Specifically, the PCE of flexible c-Si cells has continuously increased over the past three years. In this study, we achieved remarkable increases in the device size and PCE from 4 cm^2^ and 23.27% to 244.3 cm^2^ and 24.5%, respectively (Fig. 3e and Extended Data Figs. 6 and 8). The realization of industrial-size, flexible c-Si solar cells indicates that the technology route demonstrated here is compatible with standardized commercial production. At the module level, the flexible SHJ modules are free from heavy glasses and back sheets (Supplementary Figs. 8 and 9), which results in an extremely small mass-to-power ratio of 2.31 g W^−1^ that is much less than the values of 45.57 g W^−1^ and 82.93 g W^−1^ for standard monofacial and bifacial c-Si solar modules, respectively (Fig. 3f). The flexible SHJ modules demonstrated in this study may address the load-bearing issue encountered in the fast-growing research field of building-integrated photovoltaics and enable c-Si solar modules to be attached to building walls with either flat or curved surfaces.

## Operating stability

**Fig. 4:**
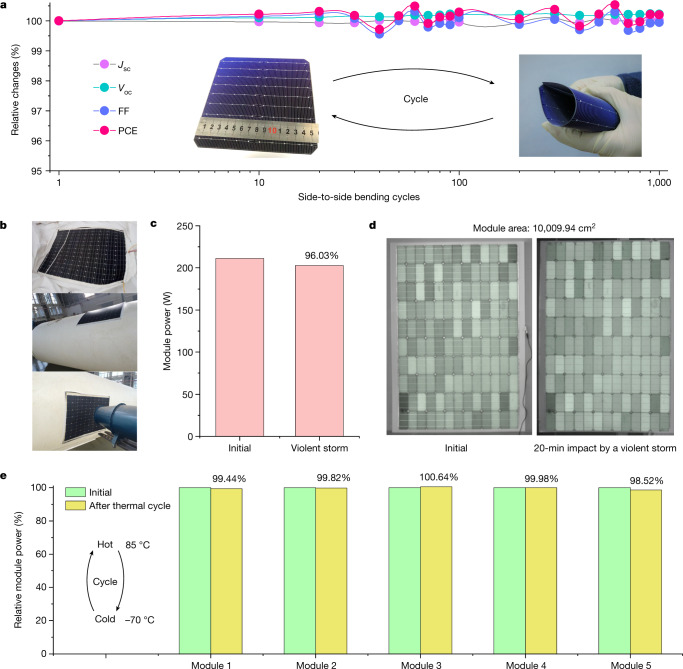
Solar cell (module) stability. **a**, Evolution of the performance of a flexible SHJ solar cell during a bending cycle. In each cycle, one edge was folded to touch the opposite edge; this bending was maintained for more than 10 s. **b**, A large (>10,000 cm^2^) flexible SHJ solar module was attached to a soft gasbag. The pressure inside the gasbag was 94.7−830 Pa greater than the atmospheric pressure. Air was blown on the module by a fan to model a violent storm of 30 m  s^−1^ for 20 min. **c**,**d**, The power of the module (**c**) and electroluminescence images (**d**) before and after continuous air impact for 20 min to model a violent storm. **e**, The relative power of five flexible SHJ modules before and after thermal cycling between −70 °C and 85 °C for 120 h. In each cycle, the modules were maintained at −70 °C for 1 h and then at 85 °C for 1 h.

Finally, we investigated the operating stability of the cell (module) under extreme conditions. The device exhibited a small *R*_b_ of approximately 8 mm (Supplementary Fig. 10). The *J*_sc_, *V*_oc_, FF and PCE of the flexible cell (Fig. 4a) retained 100% of their initial values after 1,000 side-to-side bending cycles. In each cycle, one edge was folded to touch the opposite edge; this bending was maintained for more than 10 s. The results for the bending cycles in the perpendicular direction are shown in Supplementary Fig. 11. These results were substantially different from that obtained for the flexible perovskite solar cell (Extended Data Fig. 8), in which the PCE decreased from 21% to 17% after the bending cycles. This decrease in PCE possibly originated from structural failure at grain boundaries in the polycrystalline perovskite film.

We assembled cells into a 10,009.94 cm^2^ flexible module and attached this module to an inflated gasbag. Then, we used a powerful fan to model the effect of wind blowing at a speed of 30 m s^−1^ during a violent storm^39^ (Beaufort number 11: 28.5−32.6 m s^−1^; Fig. 4b and Supplementary Video 6). After continuous air impact for 20 min, the relative power loss was only 3.07% (Fig. 4c), which was consistent with the negligible changes in the electroluminescence images (Fig. 4d). This suggested that the flexible module can robustly operate under vibration conditions, which was also validated by the vibration cycles and free-falling cycles (Supplementary Figs. 12 and 13 and Supplementary Videos 7 and 8).

The lightweight nature of the flexible SHJ modules makes them suitable for charging near-space aerial vehicles^40^, in which the temperature can reach as low as −70 °C at a height of 20−75 km. To model this, we cycled the flexible modules between −70 °C for 1 h and 85 °C for 1 h. After continuous temperature cycling for 120 h, the average relative power loss was only 0.32% (Fig. 4e), which shows that these modules can safely be operated in cold near-space conditions or on the South Pole or North Pole, as well as in deserts during hot summers.

**Extended Data Fig. 9:**
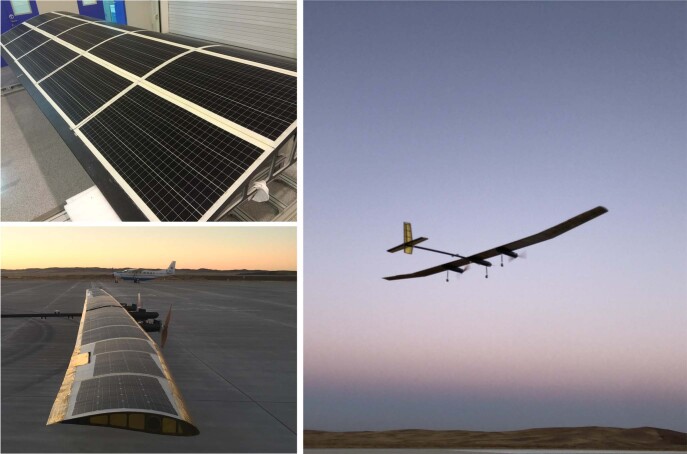
Application of flexible SHJ solar cells on a near-space unmanned aerial vehicle. We installed lightweight flexible SHJ modules on a near-space unmanned aerial vehicle that safely flew at a height of 20 km thanks to the characteristics of the SHJ modules, which included light weight, flexibility and stability in cold environments.

Although the results obtained here for our cells are promising, they are not sufficient to guarantee consistent stability under real-life operating conditions in which stressors may occur simultaneously. Thus, more in situ tests need to be performed before large-scale production. So far, we have installed our flexible SHJ modules on near-space unmanned aerial vehicles (Extended Data Fig. 9) and on the South Pole (Supplementary Fig. 14). They can steadily supply electricity under extreme conditions and the output power is sometimes greater than the designed values, which probably results from the low temperature^41^ and bifacial characteristics^42,43^.

We showed that the mechanical performance of a material is not exclusively determined by its lattice structure at the atomic level; the symmetry at the mesoscale also plays an important part. At present, we can manufacture more than 60,000 flexible SHJ cells daily with a fragmentation rate of less than 2% in our production line. This demonstrates an inexpensive strategy for commercial production of high-performance flexible c-Si solar cells. This might lead to a notable growth of the flexible cell market in the near future. Moreover, the concept demonstrated here applies, but is not limited, to solar cell fabrication; it should also be of interest to the community of researchers interested in other flexible electronics^44–46^.

## Methods

### Stress simulation

The solid mechanics module in COMSOL Multiphysics (v.5.6) was used to simulate the stress of a two-dimensional silicon wafer with the length and thickness set to 1 cm and 60 μm, respectively. The Young’s modulus, Poisson’s ratio and mass density of the wafer were 130 GPa, 0.26 and 2.33 g cm^−3^, respectively. The lower surface was textured with pyramids ranging from 5 μm to 8 μm in height and the initial angle between adjacent sharp pyramids was 71°. Three points around the midpoint of the top side of the wafer were fixed and bending forces of *F*_b_ = 1.2 mN were loaded on its two end points. The maximum von Mises stress was simulated as a function of the channel radius (*R*_p_).

### Atomistic simulation

Large-scale atomic/molecular massively parallel simulator (LAMMPS) package^47^ was used to perform atomistic simulations of mode I loading on c-Si nanofilms with sharp and round channels between surface pyramids. The Tersoff potential^48^ was used to describe the interatomic interaction between Si atoms. The simulated samples were 217.24 nm × 54.21 nm × 2.17 nm in size, containing approximately 1,150,000 Si atoms oriented along the [100], [010] and [001] directions with respect to the *x*, *y* and *z* axes, respectively. The *R*_p_ of the channels between the pyramids was increased from 0 to 15.81 nm. Periodic boundary conditions were imposed in the *y* and *z* directions of the simulation systems. Mode I loading was performed by uniformly stretching the simulation box with a strain rate of 5 × 10^8^ s^–1^. Deformation processes coloured by the von Mises shear strain as well as stress−strain curves of the simulation samples with sharp and blunt notches were recorded as videos. Cracking of the blunted sample initiated at a higher loading strain of 17.3% compared with that of 9.3% for the untreated sample. Here, the simulation was qualitative because the *R*_p_ values were much smaller than those in the experimental conditions.

### TEM characterization

An in situ bending test of a c-Si foil was conducted on an FEI Tecnai F30 TEM system using an electrical holder from PicoFemto. The c-Si foil was 6 μm × 12 μm × 70 nm in size, which was cut from the top surface of a wafer with sharp pyramids using a ThermoFisher Scios 2 FIB–SEM system, followed by deposition of a Pt film on the surface to protect the sharp pyramids. Then, the c-Si foil was welded onto a copper FIB holder with a diameter of 3 mm. A tungsten tip was used to contact the left side of the FIB c-Si foil; the movement of the foil was controlled by a piezo manipulator at a rate of about 0.01 nm s^−1^, to apply a bending force on the edge of the c-Si foil with an estimated strain rate of 10^−3^ s^−1^. For all bending processes, a 300-kV voltage with a weak electron beam was used in the TEM system to minimize the potential beam effects on the bending deformation. The real-time stress distribution was recorded by a charge-coupled device camera at a rate of 20 frames per second.

The fracture surfaces of two 60-μm wafers with sharp and round channels between pyramids were protected by a bilayer consisting of carbon and Pt films. In particular, a carbon film with a thickness of 100 nm was deposited by magnetron sputtering (ISC150 T Ion Sputter Coater) for nondestructive protection of the surface; then, the FIB-TEM foils were cut from the fracture surface using a ThermoFisher Scios 2 FIB–SEM system. STEM-HAADF observations were conducted at a depth of dozens of atoms from these fracture surfaces on an FEI Themis Z with a spherical aberration corrector for the illumination system.

### Three-point bending test

Load–vertical displacement (*F*–*D*) curves of 4 cm × 2 cm × 140 μm textured c-Si wafers were obtained using a commercial Discovery DMA 850 instrument (Supplementary Fig. 15). The marginal regions of these textured wafers were blunted in 10 vol% HF:90 vol% HNO_3_ solution for 0, 15 and 30 s.

### GPA

The elastic strain distribution in the fractured c-Si wafers was mapped using GPA on the basis of the individual high-resolution STEM images. GPA, which was done on the basis of the formalism given in the literature^17^ and implemented in the Gatan Digital Micrograph as a plug-in, was used to calculate the in-plane components of the symmetric strain tensor *ε*_*ij*_. Strain maps were plotted with respect to an internal reference lattice based on g1 = (200) and g2 = (020) using Lorentzian masks with a diameter of 0.5 nm^−1^ (in reciprocal space). The maximum and minimum strains were set in the range of 5% to −5%.

### SEM characterization

Top views, side views and fracture surfaces of c-Si wafers were observed using SEM (HITACHI, SU8020). Sharp channels between the pyramids of these wafers were blunted in 10 vol% HF:90 vol% HNO_3_ solution for 0, 10, 20, 30, 40 and 90 s. The concentrations of HF and HNO_3_ were 49% and 68%, respectively, diluted in water.

### Ultraviolet–visible–infrared light characterization

The reflectivity of c-Si wafers from 300 to 1,200 nm was characterized using a UV−VIS−IR instrument (PerkinElmer Lambda 950).

### Optical simulations

We used the electromagnetic wave module in COMSOL Multiphysics (v.5.6) to simulate the transmission, reflection and absorption spectra. A stack of a 10-nm a-Si:H layer and an 80-nm tungsten-doped indium oxide layer was coated onto a 60-μm silicon slab. This structure was surrounded by air. Three silicon slabs were simulated; their surfaces were planar, pyramidal (height: 5 μm; pyramid angle: 71°) and rounded (radius: 2 μm). For the nonplanar silicon slabs, the thickness was the average distance between their boundaries. The upper and lower boundaries of the simulation region were set as Floquet boundary conditions. The wavelength and incident angle of light were distributed from 300 to 1,200 nm and from 0° to 80°, respectively. The plane wave entered from one side of the slab. The refractive index of air was 1, whereas the refractive indices of the other materials were analysed using ellipsometry. The transmittance and reflectance, defined as the ratio of the energy of the transmitted wave and reflected wave to that of the incident wave, respectively, were obtained by integrating the Poynting vector. The absorbance of the whole structure (silicon layer) was the ratio of the dissipation energy in the whole structure (silicon layer) to the energy of the incident wave.

### Ultrahigh-speed video camera characterization

High-speed imaging of the cracking process of a 60-μm c-Si wafer with sharp pyramids was studied using a Phantom V2511 ultrafast CMOS video camera. It recorded up to 100,000 frames per second through a Leica Z16 APO long-distance microscope. The resolution was approximately 17.5 µm per pixel.

### Solar cell fabrication

Czochralski *n*-type c-Si wafers were purchased from Sichuan Yongxiang. Their thickness and electrical resistivity were 160 μm and 0.3−2.1 Ω cm, respectively. The saw damage was removed in a 20.0-vol% alkaline water solution at 80 °C and the duration was varied to obtain different wafer thicknesses. Then, the wafers were textured in a 2.1-vol% alkali water solution at 80 °C for 10 min to form microscale pyramids on the surfaces. To fabricate flexible solar cells, the approximately 2-mm-wide marginal region of these 60-μm textured wafers was blunted in 10 vol% HF:90 vol% HNO_3_ solution for 90 s at room temperature. All wafers were cleaned using a standard RCA process to remove organics and metal ions. Next, they underwent cleaning in 2.0% hydrofluoric acid water solution for 3 min to etch the surface oxide. The creative thin c-Si technology developed previously has a great potential for flexible solar cells^49,50^ because of sufficient utilization of the silicon material. Similar to the wet process, a dry method is also very efficient for improving the flexibility of the wafer (Supplementary Fig. 16). The marginal region of the wafer was blunted by a blending plasma (power, 120 W) of argon and fluorine ions for 30 min.

In a cluster plasma-enhanced chemical vapour deposition system (VHF-PECVD, IE Sunflower, OAK-DU-5; ULVAC CME-400), 5 nm *i*-a-Si:H and 15 nm *p*-a-Si:H, and 4 nm *i*-a-Si:H and 6 nm *n*-a-Si:H were deposited on the back and front sides of the wafers, respectively, in which the process temperatures were 200 ± 5 °C. The *i*-a-Si:H layers had a bilayer architecture; the first layer was grown using pure SiH_4_, whereas the second layer was grown using diluted SiH_4_ in H_2_ with a flow ratio of 1:10. A 15-s H_2_ plasma was used to improve the passivation quality at the interface of *i*-a-Si:H and *n*-c-Si. The power density, chamber pressure and gas flow ratio during deposition of the *n*-a-Si:H layer were 33 mW cm^−2^, 80 Pa and [PH_3_]:[SiH_4_]:[H_2_] = 1.5:100:1,000, respectively. The *p*-a-Si:H layer also had a bilayer architecture, for which the deposition power density, chamber pressure and gas flow ratio were 20/20 mW cm^−2^, 80/80 Pa and [B_2_H_6_]:[SiH_4_]:[H_2_] = 1:100:100/2:100:400, respectively. Tungsten-doped indium oxide was deposited by reactive plasma deposition at 150 °C and the target was 1.0% tungsten dissolved in an indium oxide target. Electrode busbars and fingers were screen-printed on the surfaces of the devices using a low-temperature silver paste, followed by two-step annealing at 150 °C for 5 min and 185 °C for 30 min. On the sides of the certified SHJ solar cells that were exposed to sunlight, fine busbars were screen-printed and a 110-nm MgF_2_ layer was deposited by electron-beam evaporation to improve the light-harvesting efficiency.

### Solar cell (module) characterization

The current–voltage characteristics of SHJ solar cells (modules) were tested with a solar simulator (Halm IV, ceitsPV-CTL2) and the light intensity was calibrated using a National Renewable Energy Laboratory reference cell. A 60-μm flexible cell was independently tested by the National Institute of Metrology in China. To compare the current density, a 140-μm brittle cell was independently tested by ISFH CalTeC in Germany. All devices were tested under a standard illumination of 100 mW cm^−2^ at 25 °C.

### Bending cycle stability

The edge of a 60-μm flexible SHJ solar cell was folded to touch the opposite edge; this bending was maintained for more than 10 s. The bending speed was approximately 1,000 mm min^−1^. The *J*_sc_, *V*_oc_, FF and PCE of this cell were tested with a solar simulator during 1,000 bending cycles under standard illumination of 100 mW cm^−2^ at 25 °C. The bending test was conducted in directions vertical and parallel to the direction of the busbars. We also monitored the sheet resistance of the 80-nm tungsten-doped indium oxide layer on a 60-μm quasiplanar c-Si substrate during 500 side-to-side bending cycles (Supplementary Fig. 17).

### Vibration experiment

A 1,260 mm × 860 mm flexible SHJ module was installed on a large vibration platform, in which the module was supported by metal holders with a height of approximately 3 cm. The module vibrated in the *z* direction, expressed as *Z*(*t*) = *Z*_0_sin(2π*t*/*T*), where the vibration amplitude *Z*_0_ = 5 mm and the vibration period *T* = 200 ms. Electroluminescence images and the power of this flexible module before and after 18,000 vibration periods were obtained.

### Free-falling test

We made a 5.4-kg, 520 mm × 520 mm module using our flexible SHJ solar cells, which was subjected 15 times to continuous free-falling from a height of approximately 500 mm. Its power was recorded before and after the free-falling cycles.

### Thermal cycling stability

Thermal cycling was conducted between −70 °C and 85 °C for 120 h. In each cycle, the module was maintained at −70 °C for 1 h and at 85 °C for 1 h.

### Violent storm stability

Flexible SHJ solar cells were encapsulated in a large (>10,000 cm^2^) module, which was attached to a large soft gasbag inflated with air to support this flexible module. The pressure inside the gasbag was 94.7−830 Pa higher than the atmospheric pressure. A powerful fan was used to blow air on the module at a wind speed of 30 m s^−1^ to model a violent storm (Beaufort number 11: 28.5−32.6 m s^−1^). The power and electroluminescence images of this module before and after continuous impact by this air flow for 20 min were obtained.

### Reporting summary

Further information on research design is available in the Nature Portfolio Reporting Summary linked to this article.

## Online content

Any methods, additional references, Nature Portfolio reporting summaries, source data, extended data, supplementary information, acknowledgements, peer review information; details of author contributions and competing interests; and statements of data and code availability are available at 10.1038/s41586-023-05921-z.

## Supplementary information

Supplementary Information This file contains Supplementary Figs. 1–17 and Supplementary Table 1.

Reporting Summary

Supplementary Notes This zipped folder contains Certificate Reports 1–3 and Vibrational Test Report. Descriptions of the four reports are also provided.

Supplementary Video 1 Shaking a flexible wafer. We can violently shake a flexible silicon wafer like a sheet of flexible paper.

Supplementary Video 2 Simulated cracking of a wafer with sharp pyramids. Atomistic simulations found the fracture initiated under a loading strain of 9.3%. The fracture surface was smooth.

Supplementary Video 3 Simulated cracking of a wafer with blunted pyramids (*R*_p_ = 5.27 nm). Atomistic simulations found the fracture initiated under a loading strain of 12.0%. The fracture surface was slightly rough.

Supplementary Video 4 Simulated cracking of a wafer with blunted pyramids (*R*_p_ = 10.54 nm). Atomistic simulations found the fracture initiated under a loading strain of 13.8%. The fracture surface became much rougher, in which jagged notches were observed along the complicated fracture path.

Supplementary Video 5 Simulated cracking of a wafer with blunted pyramids (*R*_p_ = 15.81 nm). Atomistic simulations found the fracture initiated under a loading strain of 17.3%. The fracture surface was the roughest, in which jagged notches were observed along the most tortuous fracture path.

Supplementary Video 6 Impact of a violent storm. The flexible SHJ module was blown by a violent storm.

Supplementary Video 7 Vibration cycles. The flexible SHJ module was vibrated in the vertical direction.

Supplementary Video 8 Free-falling cycles. The flexible SHJ module experienced free-falling cycles.

## Data Availability

All data generated or analysed during this study are included in the published Article and its Supplementary Information.
