# Supplementary material for: Flexible solar cells based on foldable silicon wafers with blunted edges
Source: Nature. 2023 May 24;617(7962):717–23. doi: 10.1038/s41586-023-05921-z (PMC10208971; doi:10.1038/s41586-023-05921-z)
Supplement: Supplementary file 1 — This file contains Supplementary Figs. 1–17 and Supplementary Table 1. [file 41586_2023_5921_MOESM1_ESM.pdf]

nature portfolio

https://doi.org/10.1038/s41586-023-05921-z

Supplementary information

Flexible solar cells based on foldable silicon wafers with blunted edges

In the format provided by the authors and unedited

Flexible solar cells based on foldable silicon wafers with blunted edges

Wenzhu Liu, Yujing Liu, Ziqiang Yang, Changqing Xu, Xiaodong Li, Shenglei Huang, Jianhua Shi, Junling Du, Anjun Han, Yuhao Yang, Guoning Xu, Jian Yu, Jiajia Ling, Jun Peng, Liping Yu, Bin Ding, Yuan Gao, Kai Jiang, Zhenfei Li, Yanchu Yang, Zhaojie Li, Shihu Lan, Haoxin Fu, Bin Fan, Yanyan Fu, Wei He, Fengrong Li, Xin Song, Yinuo Zhou, Qiang Shi, Guangyuan Wang, Lan Guo, Jingxuan Kang, Xinbo Yang, Dongdong Li, Zhechao Wang, Jie Li, Sigurdur Thoroddsen, Rong Cai, Fuhai Wei, Guoqiang Xing, Yi Xie, Xiaochun Liu, Liping Zhang, Fanying Meng, Zengfeng Di, Zhengxin Liu

This Supplementary Information includes:

Supplementary Figs. 1-17

Supplementary Table 1

Other Supplementary Materials for this manuscript include the following:

Extended Data Figs. 1-9

Supplementary Videos 1-8

Supplementary Certificate Reports 1-3

Supplementary Vibrational Test Report

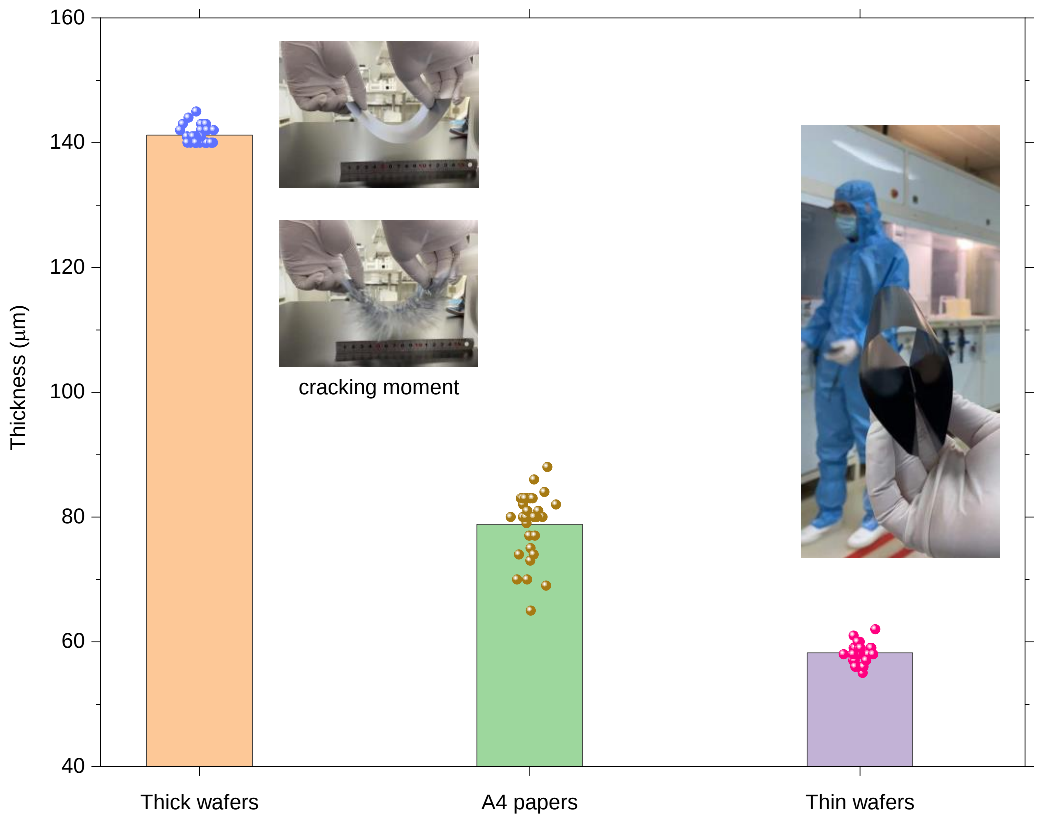

Supplementary Fig. 1 | When a 160 μm c-Si wafer is reduced to a medium thickness of 60 μm. It began to show a flexible nature resembling that of A4 papers. The boxes represent the mean values.

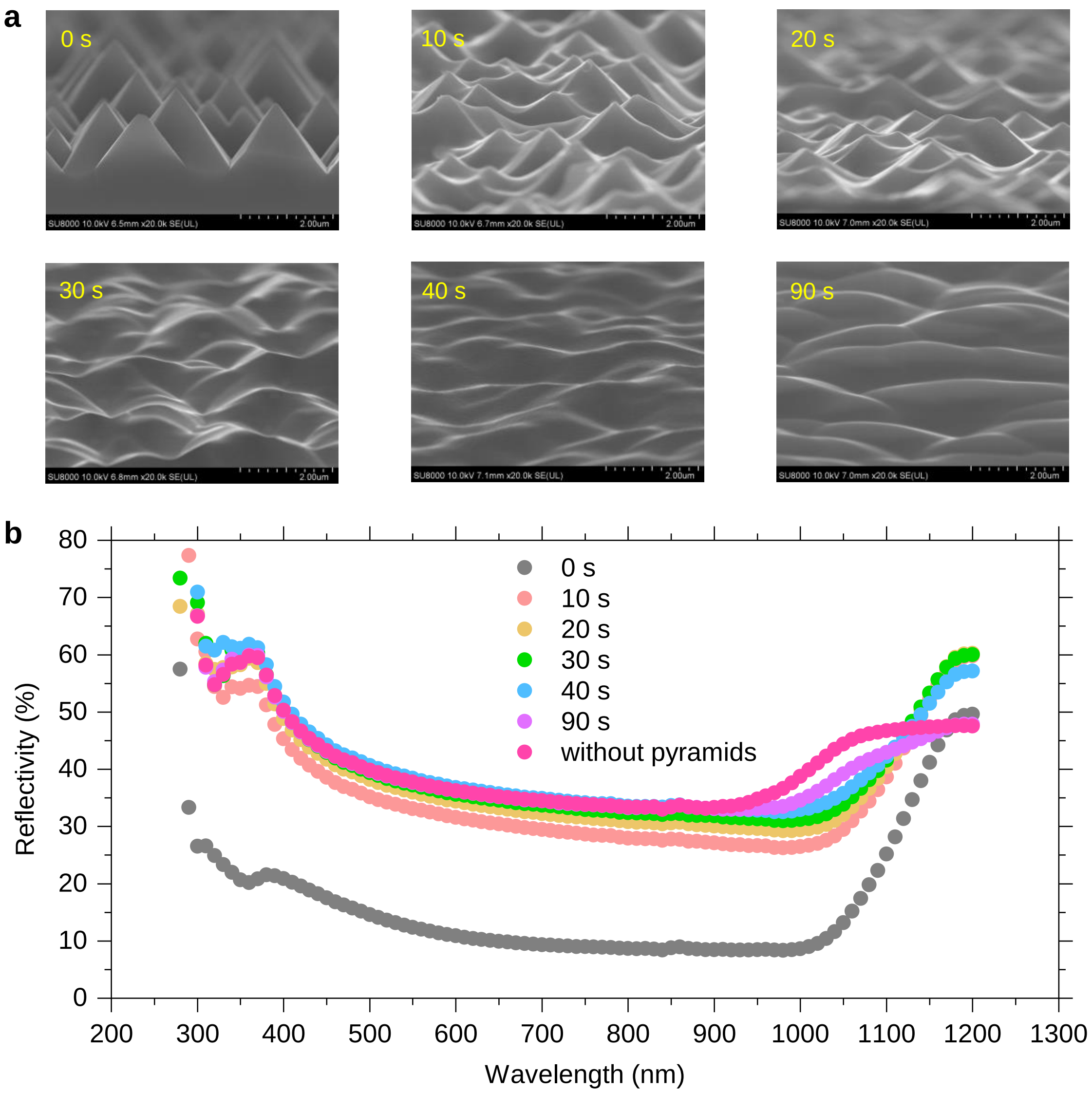

Supplementary Fig. 2 | Effect of the blunting treatment on the reflectivity of textured c-Si wafers. **a**, SEM images of surface pyramids as a function of the treatment time in 10 vol% HF/90 vol% HNO_3 solution. **b**, Their surface reflectivity was measured by a UV–VIS–NIR spectrometer. This blunting treatment significantly reduced the light-harvesting efficiency.

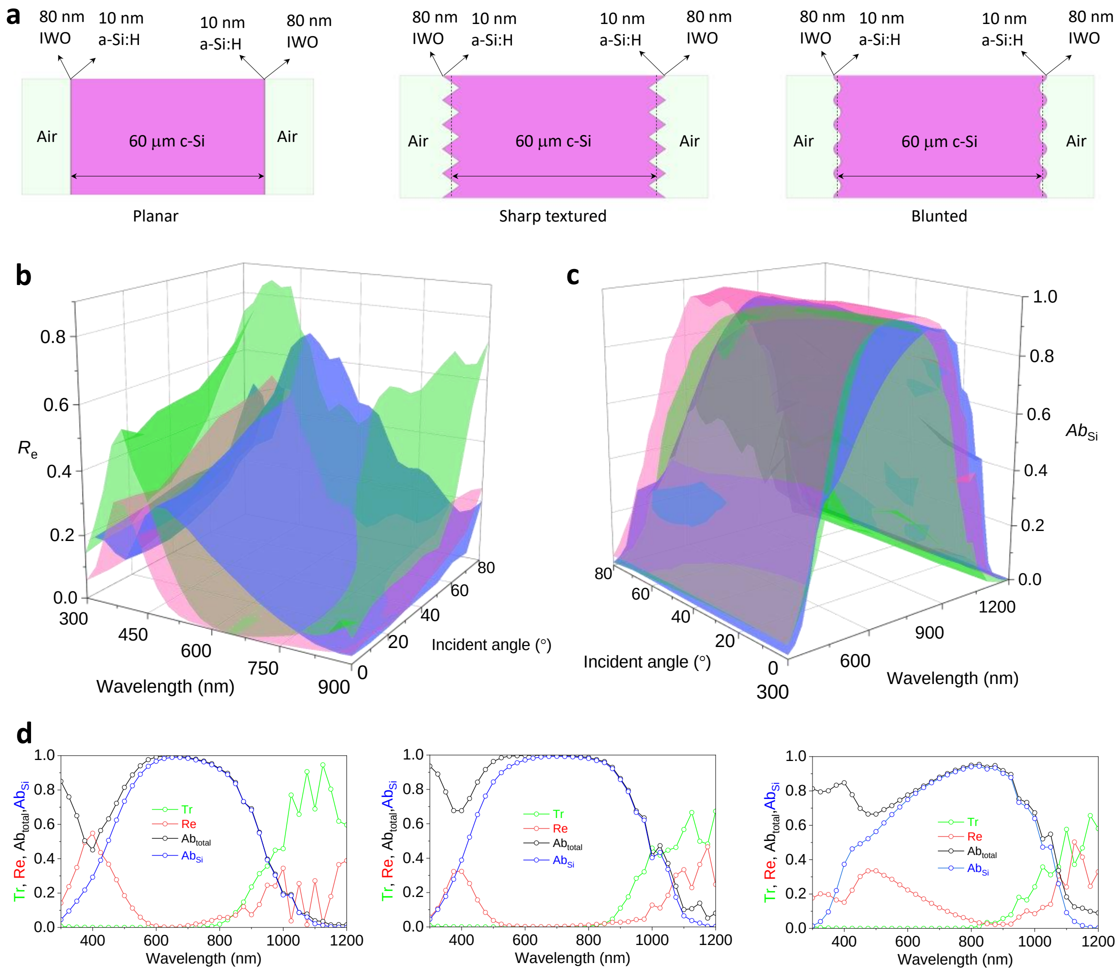

Supplementary Fig. 3 | Optical simulations of SHJ solar cells. **a**, Simulated devices with planar, sharp-pyramid and blunted surfaces (from left to right). **b**, Reflection (R_e) of the incident light as a function of wavelength and incident angle. **c**, Light trapping (Ab_{Si}) of the incident light in the c-Si absorber as a function of wavelength and incident angle. **d**, Transmittance (Tr), R_e, absorption (Ab_{total}) of the simulated devices at an incident angle of 0°, along with their Ab_{Si}. From left to right, the devices' surfaces are planar, sharp-pyramid and blunted. In **b** and **c**, the green, pink and blue colours represent the samples from left to right in **a**.

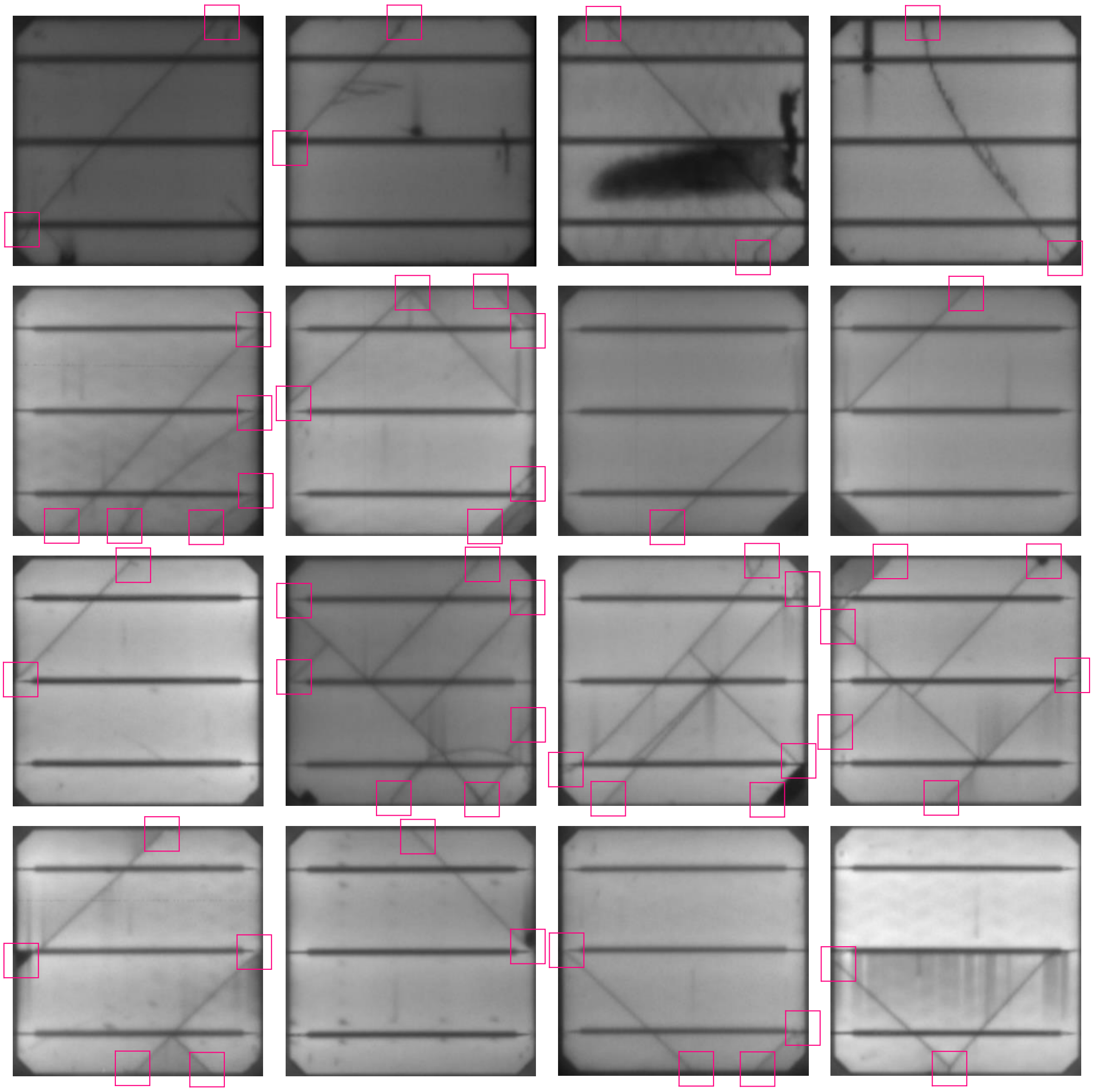

Supplementary Fig. 4 | Linear cracks on c-Si solar cells characterized by EL images. Most dark lines started from the cells' edge (red squares).

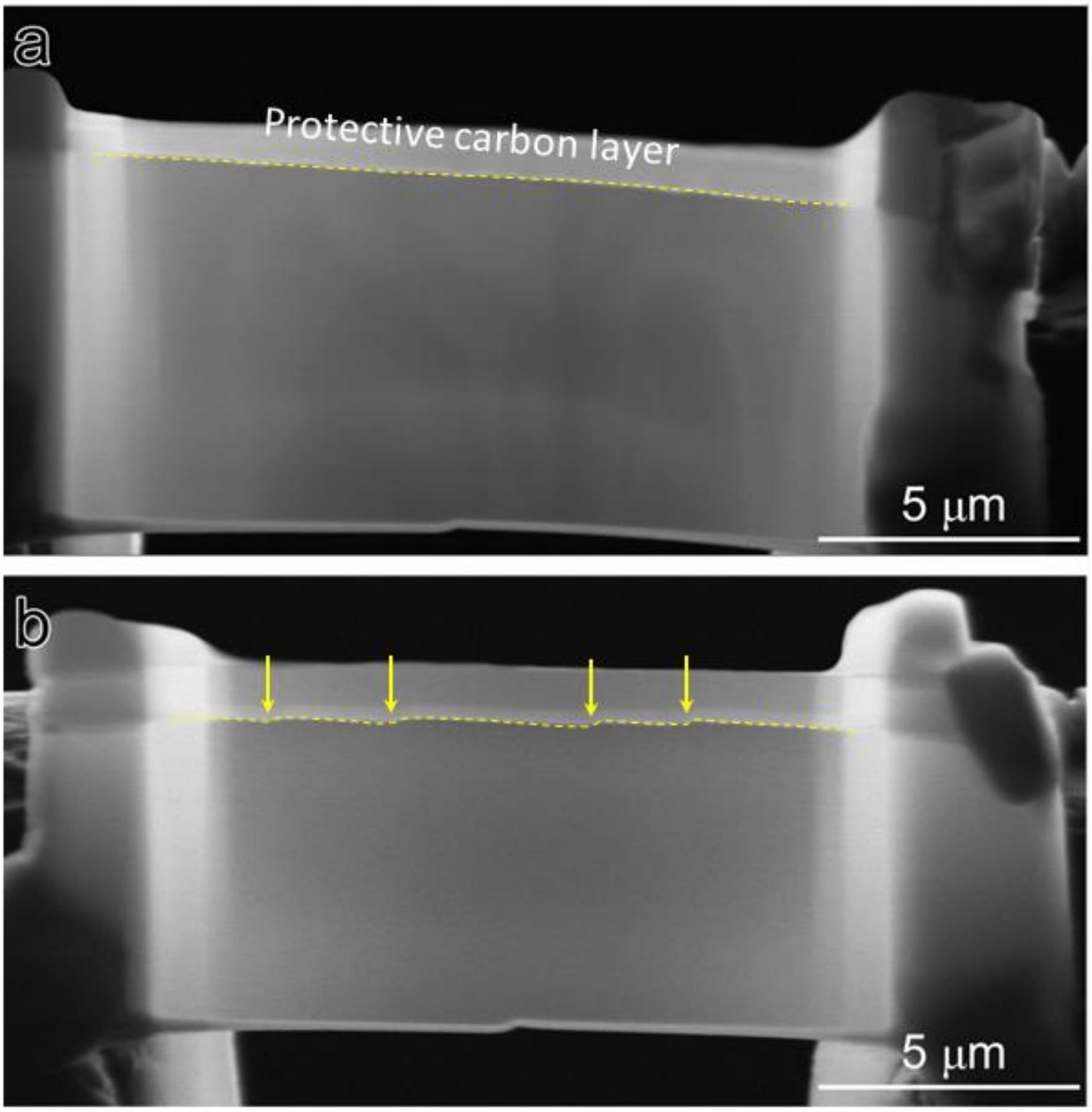

Supplementary Fig. 5 | FIB images of fracture surfaces. **a**, Side view of the fracture surface of a 60 μm c-Si wafer with sharp pyramids. **b**, Side view of the fracture surface of a 60 μm c-Si wafer whose sharp pyramids were blunted in 10 vol% HF/90 vol% HNO_3 solution for 30 s. The latter had a stepwise fracture surface with multiple cleavage sites.

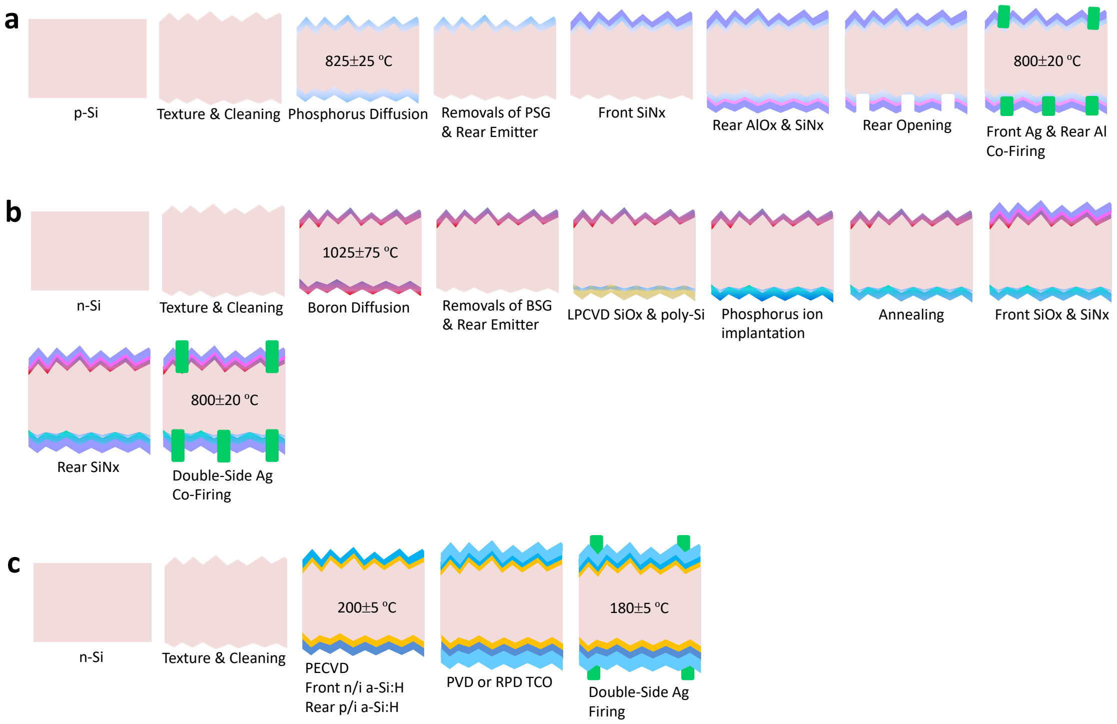

Supplementary Fig. 6 | Current fabrication of industrial c-Si solar cells. The peak temperatures of the **a**, PERC, **b**, TOPCon and **c**, SHJ solar cells were 825 ± 25 °C, 1025 ± 75 °C and 200 ± 5 °C, respectively. The firing contact between TCO layers and low-temperature Ag electrodes were realized at relatively low temperatures. Large-scale flexible manufacturing was feasible for SHJ technology because of the low process temperatures and symmetrical structural design.

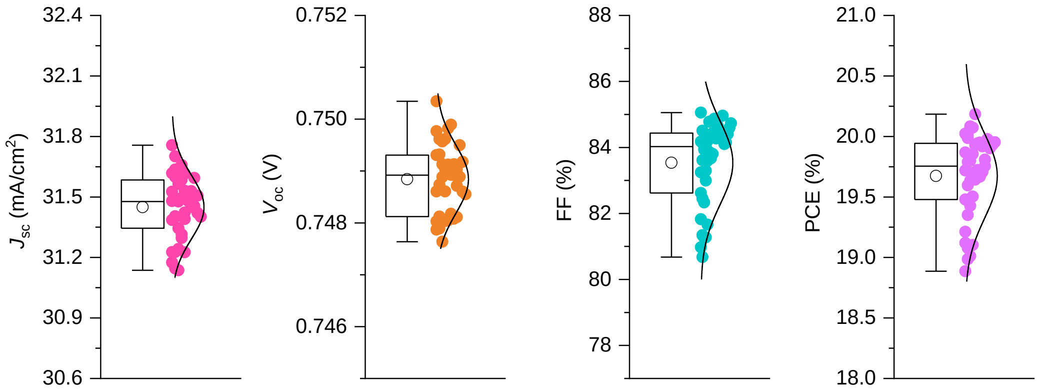

Supplementary Fig. 7 | Performance of flexible SHJ solar cells fabricated from 60 μm quasiplanar wafers, i.e., these wafers are not chemically etched to form surface pyramids. The top lines, bottom lines, lines in the box, circles and boxes represent maximum values, minimum values, median values, mean values and 25–75% distributions, respectively.

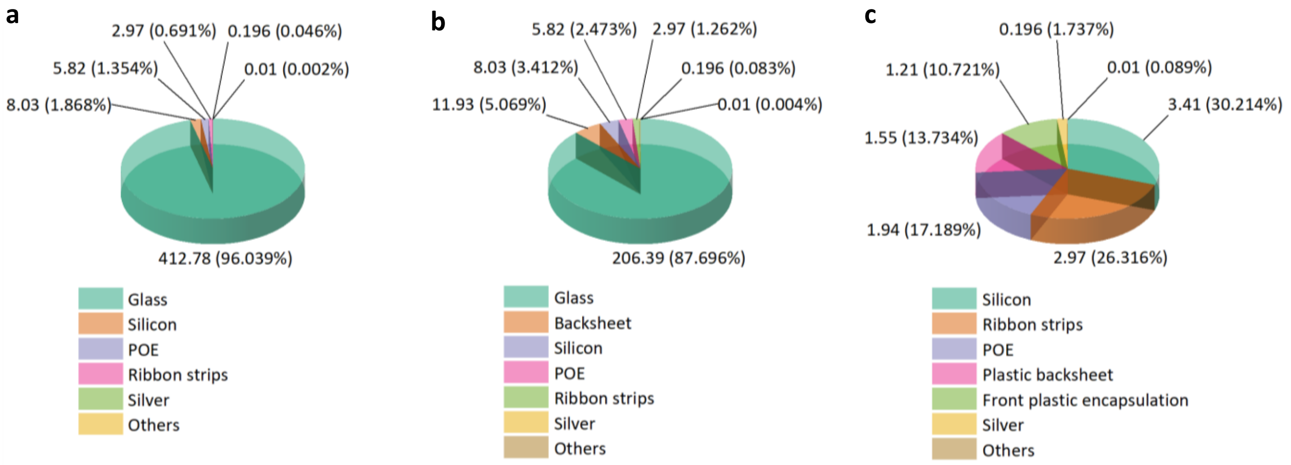

Supplementary Fig. 8 | Lightweight flexible SHJ solar modules. The bifacial and monofacial modules were assembled from 140 μm SHJ solar cells. The flexible module was assembled from 60 μm SHJ solar cells. The mass components of these mini modules are shown in **a** (bifacial), **b** (monofacial) and **c** (flexible), where the unit is grams.

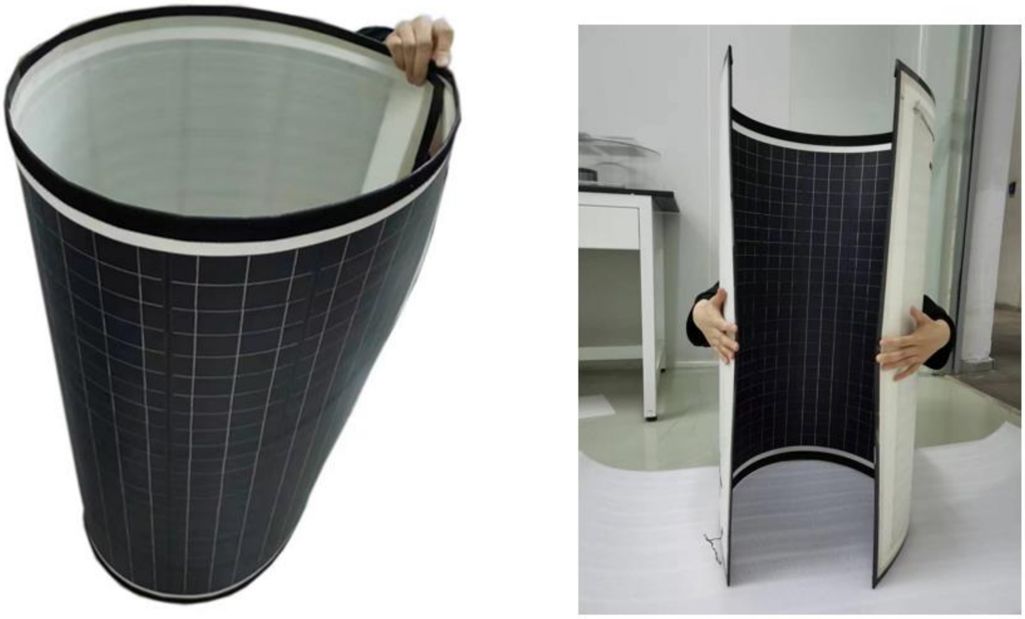

Supplementary Fig. 9 | Large module made from the flexible SHJ solar cells, featuring characteristics of light weight and rolling nature.

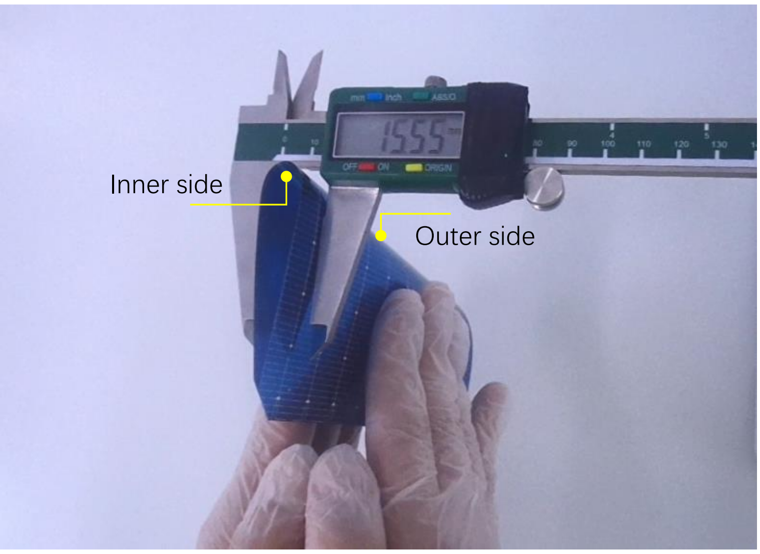

Supplementary Fig. 10 | R_b of a 6-inch SHJ solar cell tested by a vernier caliper. The inner side and the outer side of the 6-inch SHJ solar cell experienced great compressive and tensile stress respectively.

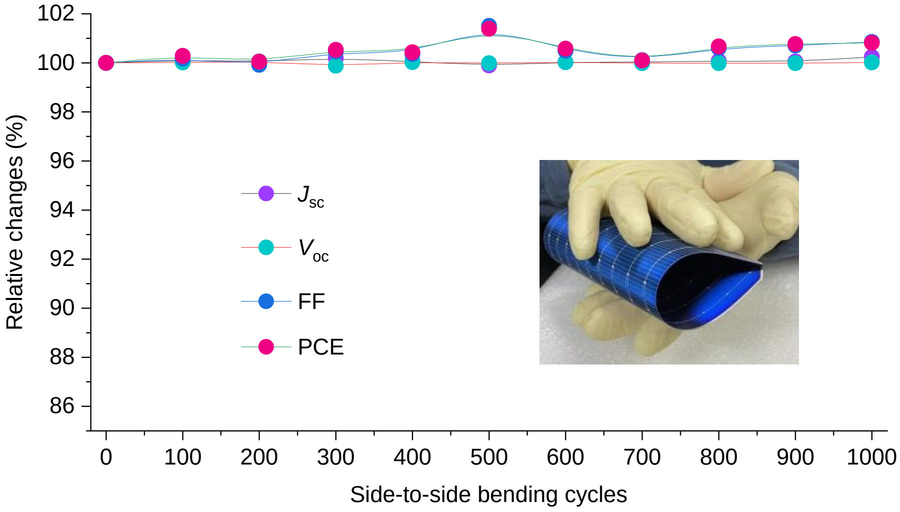

Supplementary Fig. 11 | Evolution of J_{sc}, V_{oc}, FF and PCE of a flexible SHJ solar cell during the bending cycles. The bending direction is vertical to that in Fig. 4a.

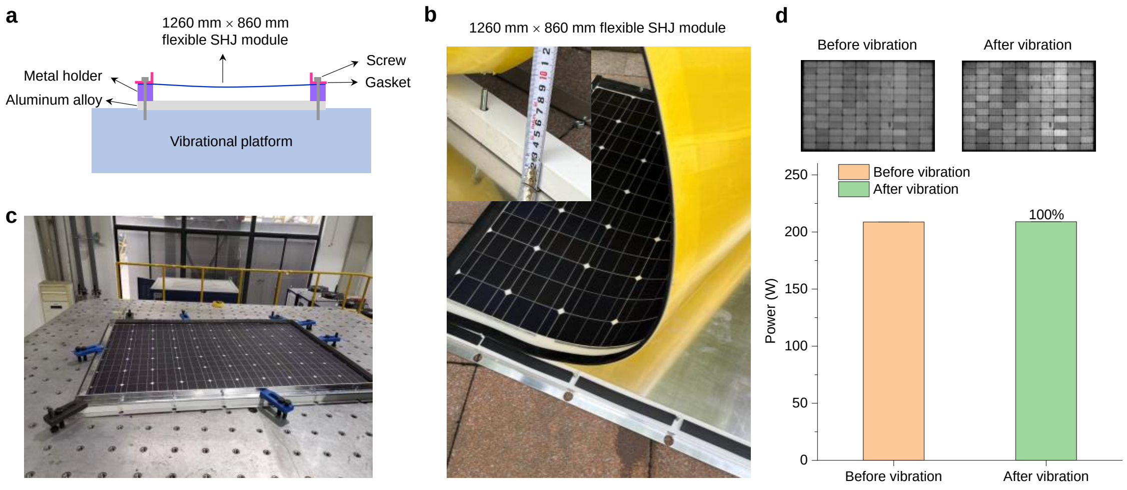

Supplementary Fig. 12 | Vibration experiment. a, Configuration of the vibrational experiment. The flexible SHJ module was installed on a large vibrational platform, where the module was supported by metal holders with a height of about 3 cm. The module vibrated in the 'z' direction, expressed as $Z(t) = Z_0 \cdot \sin(2\pi t/T)$, where the vibration amplitude $Z_0 = 5$ mm, and the vibration period $T = 200$ ms. **b**, Photo of the 1260 mm × 860 mm flexible SHJ module. **c**, Photo of the installed module on the vibrational platform. **d**, EL images and powers of this flexible module before and after 18,000 vibration periods.

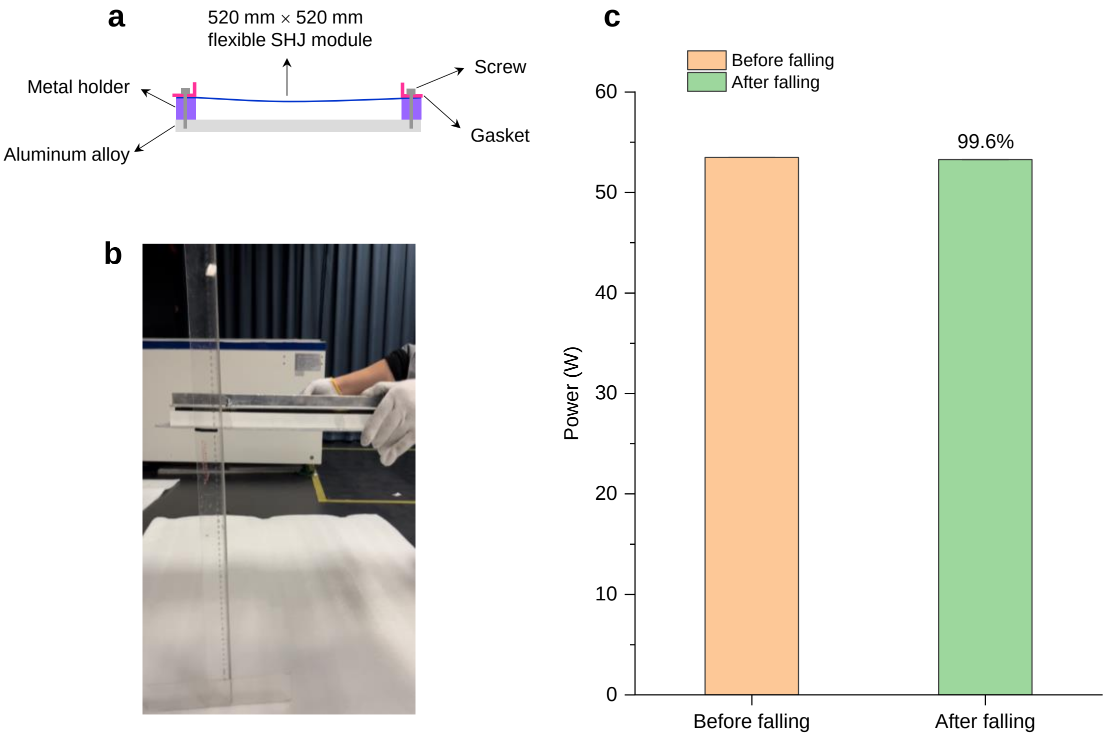

Supplementary Fig. 13 | Free-falling test. **a**, Configuration of the 520 mm × 520 mm module, whose mass is about 5.4 kg. **b**, It freely fell from a height of about 500 mm for 15 times, during which the flexible module vibrated violently. **c**, Power of the module before and after the 15 free-falling cycles.

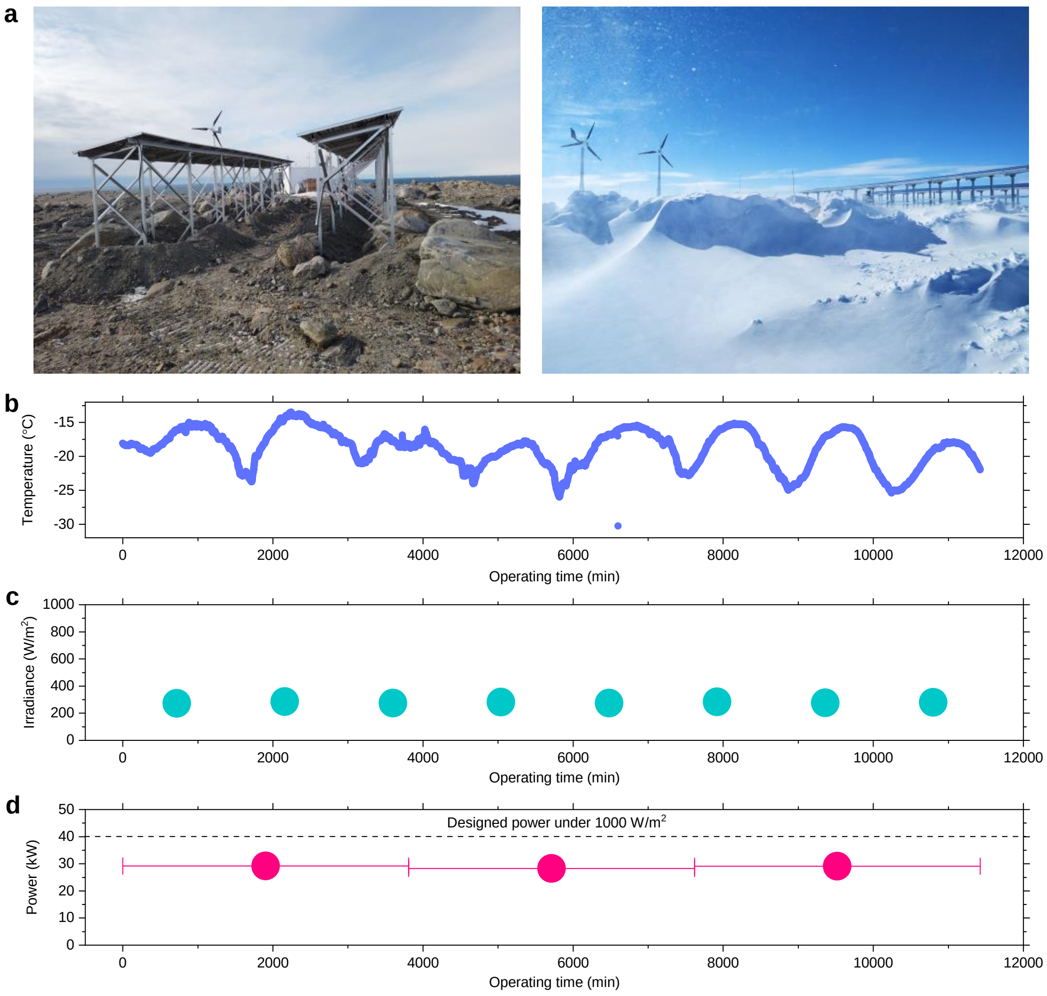

Supplementary Fig. 14 | Our bifacial SHJ solar modules were deployed at the South Pole; they were clamped in hard brackets to withstand frequent strong winds. The data are collected over the duration of polar days. **a**, Photographs of the system made from SHJ modules. **b–d**, Environmental temperatures, average solar irradiance and average power generation of these bifacial SHJ modules. They produced approximately 30 kW of electricity under an irradiance of approximately 280 W·m^{-2}. Considering that the designed power was 40 kW under a standard illumination of 1000 W·m^{-2} at 25 °C, 30 kW under real operating conditions exceeded our expectations. This superior performance probably resulted from the high bifaciality of SHJ solar cells and high albedo by snow on the ground.

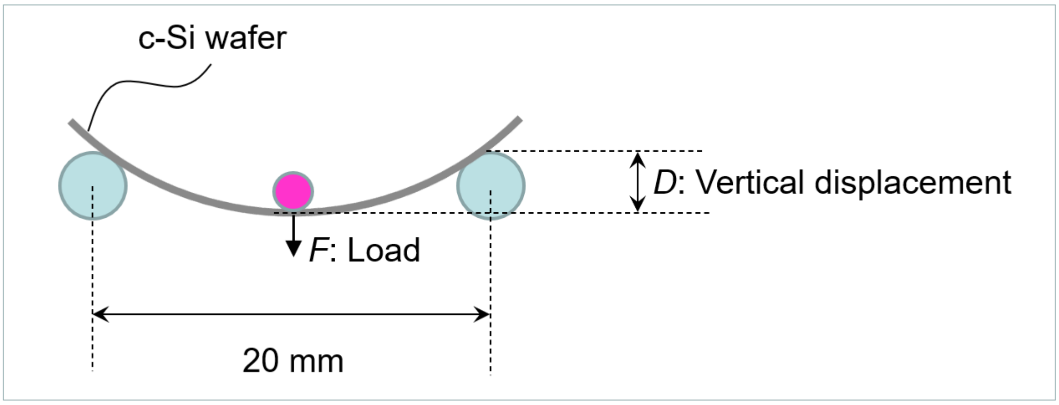

Supplementary Fig. 15 | Schematic of the Discovery DMA 850 for three-point bending test. The c-Si wafer is put onto two parallel fixed poles (light blue). Another free pole (pink) moves in the ‘z’ direction to bend the wafer. The force (*F*) and the vertical displacement (*D*) are recorded during the bending test.

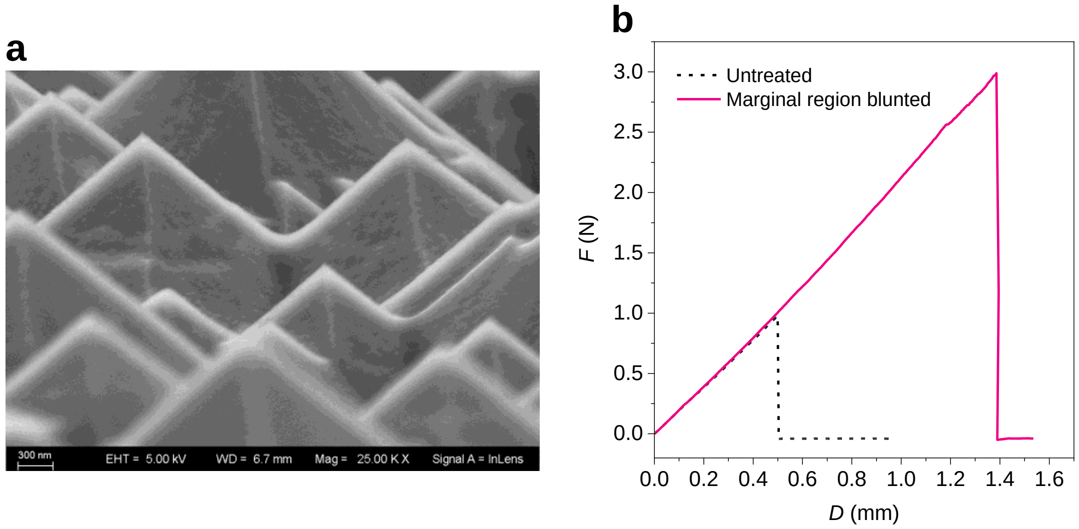

Supplementary Fig. 16 | Dry blunting treatment. We cut two pieces of 4 cm × 1.0 cm textured wafers from one large wafer with the same orientation. One was kept as the initial state (dashed line), while marginal region of the other wafer was blunted by a blending plasma of argon and fluorine ions for 30 min (solid line). The *F* - *D* curves indicate this dry treatment is effective to improve the wafer's flexibility.

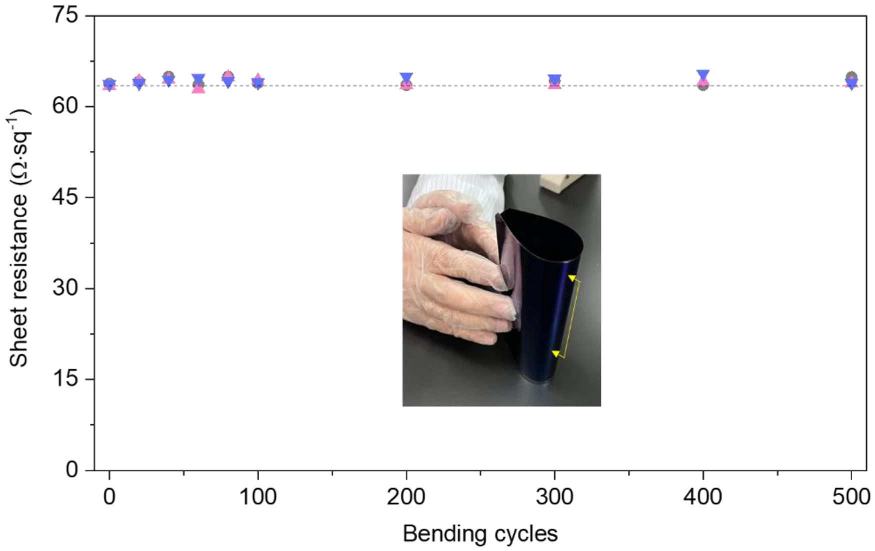

Supplementary Fig. 17 | Evolution of sheet resistance. An 80 nm IWO layer was coated on a 60 μm 15.6 cm × 15.6 cm quasiplanar c-Si substrate. Since the IWO material is much more conductive than the c-Si substrate, the test values were mainly contributed by the IWO layer. The test region is indicated by the yellow line, evidently, it exhibited a good stability under side-to-side bending cycles.

Supplementary Table 1 | Performance of flexible SHJ solar cells fabricated from quasiplanar and textured wafers.

	Quantity	Thickness (μm)	J_{sc} (mA·cm^{-2})	V_{oc} (V)	FF (%)	PCE (%)
Quasiplanar	39	60	31.45 ± 0.16	0.749 ± 0.001	83.53 ± 1.24	19.67 ± 0.34
Textured	13	55	37.59 ± 0.11	0.753 ± 0.001	82.51 ± 0.39	23.35 ± 0.13
Textured	26	65	37.65 ± 0.09	0.752 ± 0.002	82.40 ± 0.99	23.31 ± 0.33
