# Supplementary material for: Flexible solar cells based on foldable silicon wafers with blunted edges
Source: Nature. 2023 May 24;617(7962):717–23. doi: 10.1038/s41586-023-05921-z (PMC10208971; doi:10.1038/s41586-023-05921-z)
Supplement: Supplementary file 2 — Reporting Summary [file 41586_2023_5921_MOESM2_ESM.pdf]

nature research

Corresponding author(s): Wenzhu Liu, Xiaochun Liu, Liping Zhang, Fanying Meng, Zengfeng Di, Zhengxin Liu,

Solar Cells Reporting Summary

Nature Research wishes to improve the reproducibility of the work that we publish. This form is intended for publication with all accepted papers reporting the characterization of photovoltaic devices and provides structure for consistency and transparency in reporting. Some list items might not apply to an individual manuscript, but all fields must be completed for clarity.

For further information on Nature Research policies, including our data availability policy, see Authors & Referees.

Experimental design

Please check: are the following details reported in the manuscript?

1. Dimensions

Item	Yes	No	Response
Area of the tested solar cells	☒	☐	15.6 cm x 15.6 cm
Method used to determine the device area	☒	☐	The area is provided by certificate test center

2. Current-voltage characterization

Item	Yes	No	Response
Current density-voltage (J-V) plots in both forward and backward direction	☐	☒	Forward direction is enough for our silicon cells
Voltage scan conditions<br>*For instance: scan direction, speed, dwell times*	☒	☐	Forward direction from -0.1 V to 0.78 V; 100 points; integral time 5 ms; delay time 5 ms
Test environment<br>*For instance: characterization temperature, in air or in glove box*	☒	☐	Room temperature in air
Protocol for preconditioning of the device before its characterization	☐	☒	*Explain why this information is not reported/not relevant.*
Stability of the J-V characteristic<br>*Verified with time evolution of the maximum power point or with the photocurrent at maximum power point; see ref. 7 for details.*	☒	☐	Fig. 4, Supplementary Fig. 11, Supplementary Fig. 12, Supplementary Fig. 13

3. Hysteresis or any other unusual behaviour

Item	Yes	No	Response
Description of the unusual behaviour observed during the characterization	☐	☒	Silicon cells do not exhibit hysteresis under standard conditions
Related experimental data	☐	☒	Silicon cells do not exhibit hysteresis under standard conditions

4. Efficiency

Item	Yes	No	Response
External quantum efficiency (EQE) or incident photons to current efficiency (IPCE)	☐	☒	*Explain why this information is not reported/not relevant.*
A comparison between the integrated response under the standard reference spectrum and the response measure under the simulator	☐	☒	Certificate reports do not contains this comparison
For tandem solar cells, the bias illumination and bias voltage used for each subcell	☐	☒	They are not tandem solar cells

5. Calibration

Item	Yes	No	Response
Light source and reference cell or sensor used for the characterization	☒	☐	A NREL cell in 'Methods'
Confirmation that the reference cell was calibrated and certified	☒	☐	A certified NREL reference cell

Calculation of spectral mismatch between the reference cell and the devices under test

☐ Yes ☒ No

Certificate report does not contains this mismatch comparison

6. Mask/aperture

Size of the mask/aperture used during testing

☒ Yes ☐ No

The cell tested in ISFH is measured using a designated-area mode. An aperture mask (ISFH identifier FN018) shadowing the cell's edges. The test areas are larger than 226 cm2.

Variation of the measured short-circuit current density with the mask/aperture area

☐ Yes ☒ No

We did not request a full-area test in the certificate center

7. Performance certification

Identity of the independent certification laboratory that confirmed the photovoltaic performance

☒ Yes ☐ No

Extended Data Figs. 6 and 7. Full reports are found as source data along with the publication

A copy of any certificate(s)
Provide in Supplementary Information

☒ Yes ☐ No

Full reports are found as source data along with the publication

8. Statistics

Number of solar cells tested

☒ Yes ☐ No

78 cells and many other cells encapsulated in large-scale modules

Statistical analysis of the device performance

☒ Yes ☐ No

Fig. 3c

9. Long-term stability analysis

Type of analysis, bias conditions and environmental conditions
For instance: illumination type, temperature, atmosphere humidity, encapsulation method, preconditioning temperature

☒ Yes ☐ No

Fig. 4: bending cycles, thermal cycles, violent storm impact; Supplementary Fig. 14: Operation in the Antarctic Pole
