# Supplementary material for: Flexible solar cells based on foldable silicon wafers with blunted edges
Source: Nature. 2023 May 24;617(7962):717–23. doi: 10.1038/s41586-023-05921-z (PMC10208971; doi:10.1038/s41586-023-05921-z)
Supplement: Supplementary file 3 — This zipped folder contains Certificate Reports 1–3 and Vibrational Test Report. Descriptions of the four reports are also provided. [file 41586_2023_5921_MOESM3_ESM.zip › Supplementary Reports/Vibration test TUV.pdf]

Test Report

Report No. TC.22.11.006217

Date of Issue 11/15/2022

Applicant: **Shanghai Institute of Microsystem and Information Technology. Chinese Academy of Sciences**

Applicant address: **235 Chengbei Road, Jiading, Shanghai**

Description of the test subject:

Sample	Description	Photo
001	Sample Description: Flexible pv module<br><br>Style No.: A7<br><br>Manufacturer: SIMIT	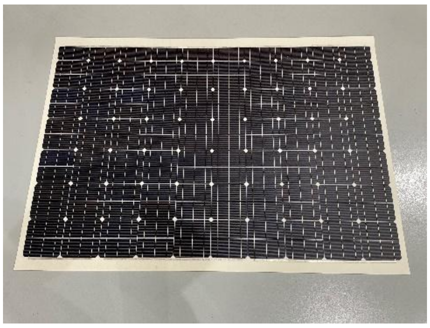

Receipt Date of Sample: **11/14/2022**

Date of Testing: **From 11/14/2022 to 11/14/2022**

Sample submitted: **The sample(s) was (were) submitted by applicant and identified.**

Conclusion:

Test Items			Result
No.	Items	Standard	
1	Vibration tests	Client's requirement	Pass

Note: (1) The TÜV SÜD SW Rail Transportation Technology (Jiangsu) Co., Ltd. General Terms & Conditions applied, for full content please visit https://www.tuvsud.cn/zh-cn/terms-and-conditions. (2) The results relate only to the sample(s) as received. (3) The test report shall not be reproduced except in full without the written approval of the company.

Laboratory:
TÜV SÜD SW Rail Transportation Technology (Jiangsu) Co., Ltd.

Phone: +86/ (0) 519- 8123-9872
Fax: +86/ (0) 519- 8123-9872 ext.123
E-mail: czx.qm@tuvsud.com
www.tuvsud.com

Regd. Office:
Innovative Industry Park, No. 377 Wuyinan Road, National High and New Technology & Industry Development Zone, Wujin, 213015 P.R. China

检验检测专用章
Inspection&Testing Stamp

Test Results

1. Vibration tests

1.1 Test equipment &Sensor installation

Test method: Client’s requirement
Vibration type: LI555555

Test environment: 19.4℃, 50%RH
Sensor number and sensitivity: 1# Sensor 29.73pC/g, 2# Sensor 27.99pC/g
Test equipment: 35T Electric vibration table IPA540H/H3580A

Sample and sensor installation:
The sample is installed on the tooling and fixed on the vibration table with a pressure plate. Two fixed points of the tooling are selected and a sensor is pasted beside them. The vibration adopts 2-point average control mode. The installation position of the sensor is shown below:

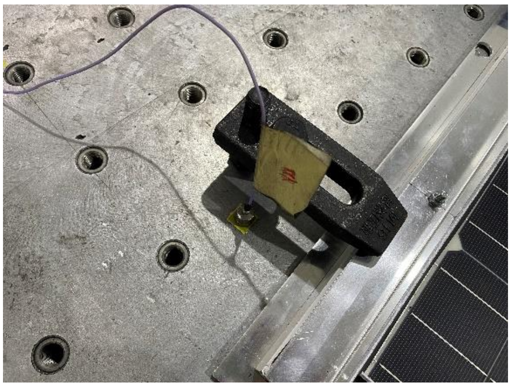 Control sensor-1	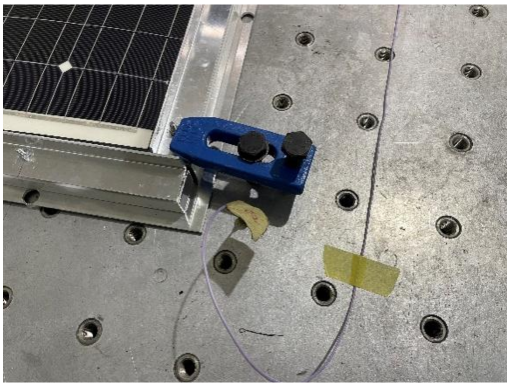 Control sensor-2

1.2 Test results

Sample No.	Direction	Sequence	Amplitude	Duration	Requirement	Results	Conclusion
TC.22.11. 006217	Vertical	5Hz	5mm	1h	No damage to appearance	No damage to appearance	Pass

Note: (1) The TÜV SÜD SW Rail Transportation Technology (Jiangsu) Co., Ltd. General Terms & Conditions applied, for full content please visit https://www.tuvsud.cn/zh-cn/terms-and-conditions. (2) The results relate only to the sample(s) as received. (3) The test report shall not be reproduced except in full without the written approval of the company.

Laboratory:
TÜV SÜD SW Rail Transportation Technology (Jiangsu) Co., Ltd.

Phone: +86/ (0) 519- 8123-9872
Fax: +86/ (0) 519- 8123-9872 ext.123
E-mail: czx.qm@tuvsud.com
www.tuvsud.com

Regd. Office:
Innovative Industry Park, No. 377 Wuyinan Road, National High and New Technology & Industry Development Zone, Wujin, 213015 P.R. China

Test Report

Report No. TC.22.11.006217

Date of Issue 11/15/2022

Test photos:

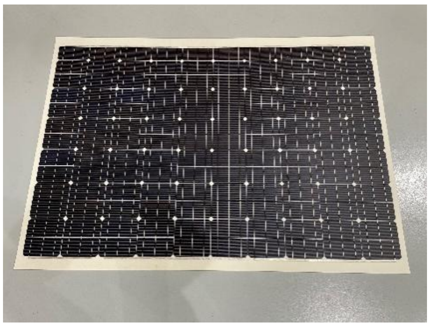

Before the test

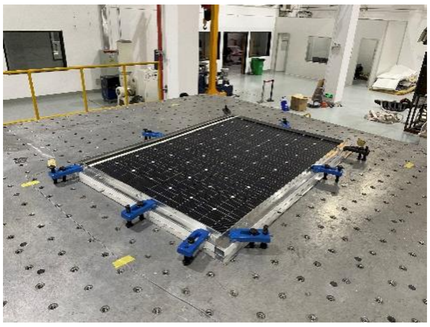

During the test

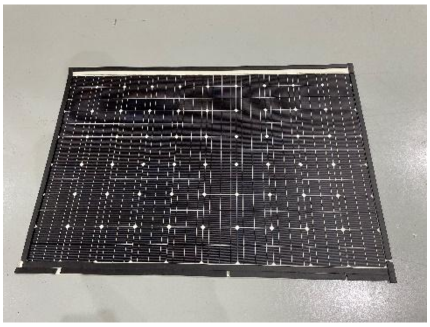

After the test

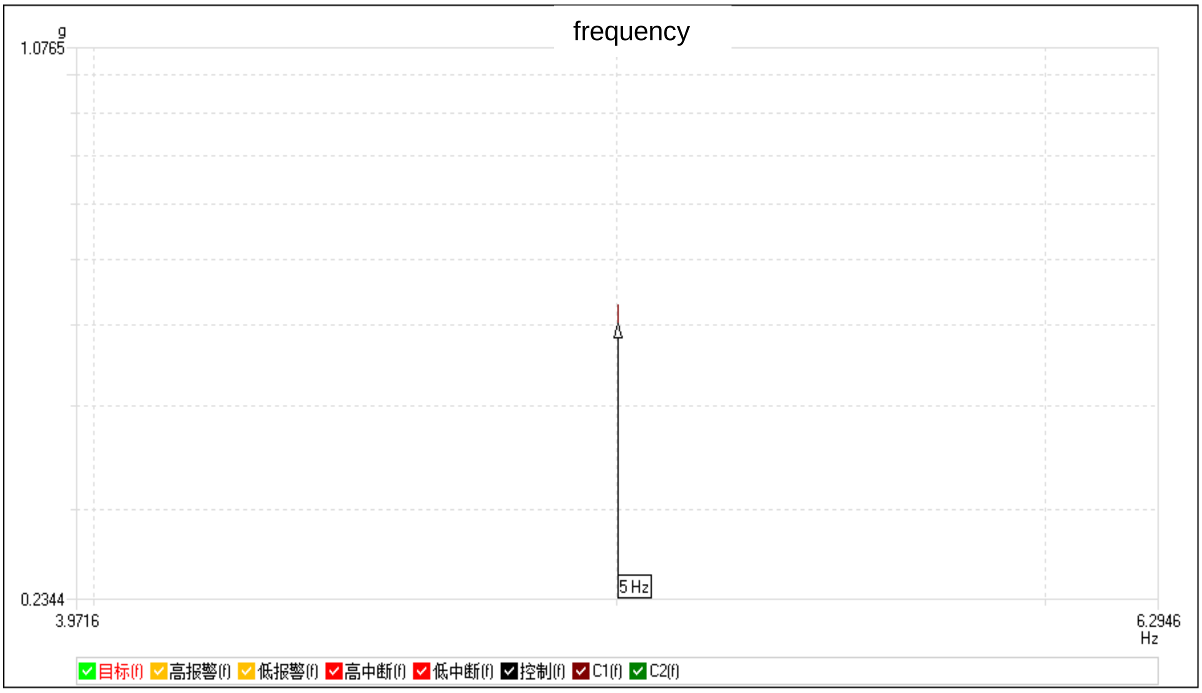

Test curve

Note: (1) The TÜV SÜD SW Rail Transportation Technology (Jiangsu) Co., Ltd. General Terms & Conditions applied, for full content please visit https://www.tuvsud.cn/zh-cn/terms-and-conditions. (2) The results relate only to the sample(s) as received. (3) The test report shall not be reproduced except in full without the written approval of the company.

Laboratory:
TÜV SÜD SW Rail Transportation Technology (Jiangsu) Co., Ltd.

Phone: +86/ (0) 519- 8123-9872
Fax: +86/ (0) 519- 8123-9872 ext.123
E-mail: czx.qm@tuvsud.com
www.tuvsud.com

Regd. Office:
Innovative Industry Park, No. 377 Wuyinan Road, National High and New Technology & Industry Development Zone, Wujin, 213015 P.R. China

Test Report

Report No. **TC.22.11.006217**

Date of Issue **11/15/2022**

Test results are just for internal reference.

TÜV SÜD SW Rail Transportation Technology (Jiangsu) Co., Ltd.

Drafted by: Approved by:

Wu Mengqi Qin Jianzhong

Mengqi Wu Jianzhong Qin

-End of Report-

Note: (1) The TÜV SÜD SW Rail Transportation Technology (Jiangsu) Co., Ltd. General Terms & Conditions applied, for full content please visit https://www.tuvsud.cn/zh-cn/terms-and-conditions. (2) The results relate only to the sample(s) as received. (3) The test report shall not be reproduced except in full without the written approval of the company.

Laboratory:
TÜV SÜD SW Rail Transportation Technology (Jiangsu) Co., Ltd.

Phone: +86/ (0) 519- 8123-9872
Fax: +86/ (0) 519- 8123-9872 ext.123
E-mail: czx.qm@tuvsud.com
www.tuvsud.com

Regd. Office:
Innovative Industry Park, No. 377 Wuyinan Road, National High and New Technology & Industry Development Zone, Wujin, 213015 P.R. China

检验检测专用章
Inspection&Testing Stamp
