# Supplementary material for: Flexible solar cells based on foldable silicon wafers with blunted edges
Source: Nature. 2023 May 24;617(7962):717–23. doi: 10.1038/s41586-023-05921-z (PMC10208971; doi:10.1038/s41586-023-05921-z)
Supplement: Supplementary file 3 — This zipped folder contains Certificate Reports 1–3 and Vibrational Test Report. Descriptions of the four reports are also provided. [file 41586_2023_5921_MOESM3_ESM.zip › Supplementary Reports/Supplementary Report descriptions.docx]

**Certificate Report 1**

Certificate report of a flexible SHJ solar cell. The IV data was independently tested in National Institute of Metrology (China).

**Certificate Report 2**

Certificate report of a SHJ solar cell with the bifacial structure. The IV data was independently tested in Calibration and Test Center (CalTeC), Solar CellsInstitut für Solarenergieforschung GmbH (Germany).

**Certificate Report 3**

Certificate report of a SHJ solar cell with an Ag reflector on the rear side. The IV data was independently tested in Calibration and Test Center (CalTeC), Solar CellsInstitut für Solarenergieforschung GmbH (Germany).

**Vibrational Test Report**

Vibrational test of a flexible SHJ solar module. The module was vibrated in TUV SUD Rail Transportation Technology (Jiangsu) Co., Ltd.
