# Supplementary material for: Flexible solar cells based on foldable silicon wafers with blunted edges
Source: Nature. 2023 May 24;617(7962):717–23. doi: 10.1038/s41586-023-05921-z (PMC10208971; doi:10.1038/s41586-023-05921-z)
Supplement: Supplementary file 3 — This zipped folder contains Certificate Reports 1–3 and Vibrational Test Report. Descriptions of the four reports are also provided. [file 41586_2023_5921_MOESM3_ESM.zip › Supplementary Reports/Certificate report 2.pdf]

Calibration Certificate

Kalibrierschein

issued by the calibration laboratory
erstellt durch das Kalibrierlaboratorium

Calibration and Test Center (CalTeC) Solar Cells
Institut für Solarenergieforschung GmbH
Am Ohrberg 1
31860 Emmerthal
GERMANY

http://caltec.isfh.de/
solarcells@caltec.isfh.de

Deutsche Akkreditierungsstelle
D-K-18657-01-00

Calibration mark
Kalibrierzeichen

003027
D-K-18657-01-00
2022-11

Object<br>*Gegenstand*	**Solar cell on gold-coated chuck**
Manufacturer<br>*Hersteller*	**Zhongwei New Energy (Chengdu) Co.Ltd.<br>Chengdu City, Sichuan Province, CHINA**
Type<br>*Typ*	**Bifacial H-pattern solar cell<br>with 9 busbars (M2)**
Serial number<br>*Fabrikat/Serien-Nr.*	**2209ZW925B-4 (DA3mm)**
Customer<br>*Auftraggeber*	**Zhongwei New Energy (Chengdu) Co.Ltd.<br>Chengdu City, Sichuan Province, CHINA**
Order No.<br>*Auftragsnummer*	**12822Z_04**
Date of calibration<br>*Datum der Kalibrierung*	**November 10, 2022**
Place of calibration<br>*Ort der Kalibrierung*	**ISFH CalTeC,<br>31860 Emmerthal, GERMANY**
Number of pages of the certificate<br>*Anzahl der Seiten des Kalibrierscheins*	**7**

This calibration certificate documents the traceability to national standards, which realize the units of measurement according to the International System of Units (SI).
The DAkkS is signatory to the multilateral agreements of the European co-operation for Accreditation (EA) and of the International Laboratory Accreditation Cooperation (ILAC) for the mutual recognition of calibration certificates.
The user is obliged to have the object recalibrated at appropriate intervals.

Dieser Kalibrierschein dokumentiert die Rückführung auf nationale Normale zur Darstellung der Einheiten in Übereinstimmung mit dem Internationalen Einheitensystem (SI).
Die DAkkS ist Unterzeichner der multilateralen Übereinkommen der European co-operation for Accreditation (EA) und der International Laboratory Accreditation Cooperation (ILAC) zur gegenseitigen Anerkennung der Kalibrierscheine.
Für die Einhaltung einer angemessenen Frist zur Wiederholung der Kalibrierung ist der Benutzer verantwortlich.

This calibration certificate may not be reproduced other than in full except with the permission of the issuing laboratory. Calibration certificates with the full name of the responsible person are valid without signature.
Dieser Kalibrierschein darf nur vollständig und unverändert weiterverbreitet werden. Auszüge oder Änderungen bedürfen der Genehmigung des ausstellenden Kalibrierlaboratoriums. Kalibrierscheine sind bei Nennung des für die Freigabe Verantwortlichen in Klarschrift auch ohne Unterschrift gültig.

Date of issue<br>*Datum der Ausstellung*	Deputy head of the calibration laboratory<br>*Stellvertretender Leiter des Kalibrierlaboratoriums*	Person in charge<br>*Bearbeiter*
Institut für Solarenergieforschung GmbH ISFH CalTeC		
November 15, 2022	Dr. David Hinken	Tobias Gandy

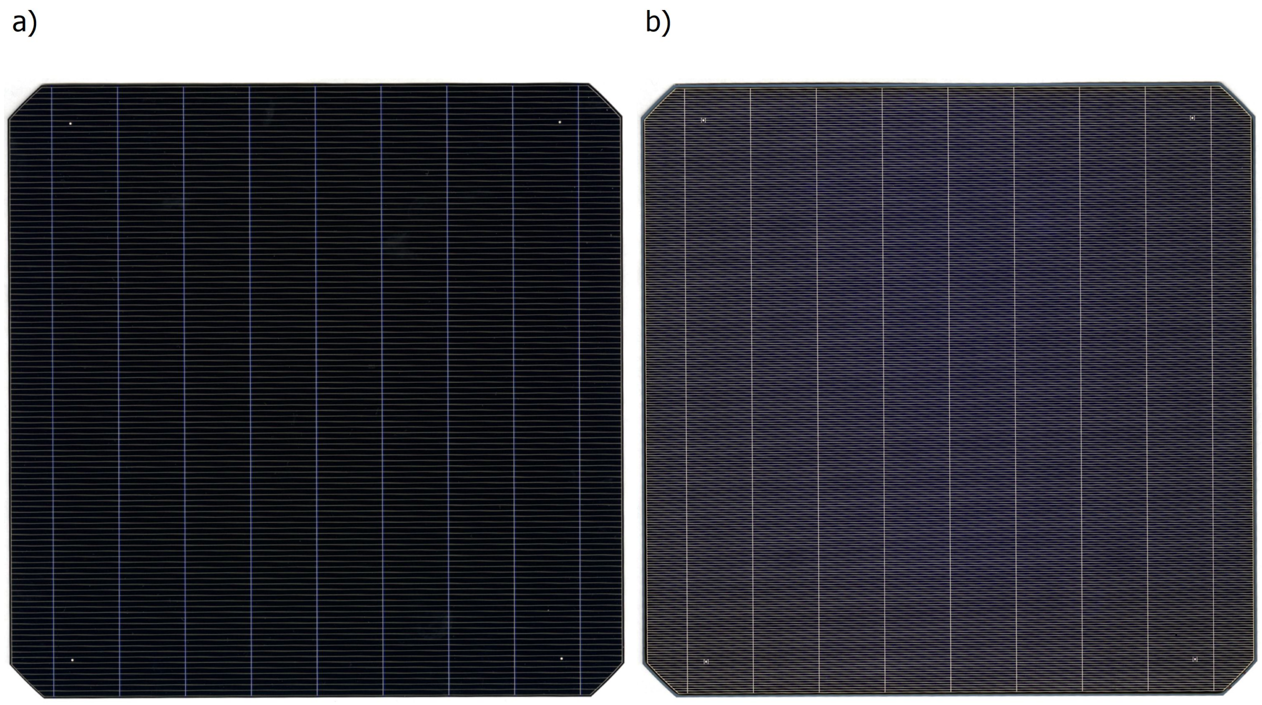

Fig. 1: Photograph of the a) front and b) rear side of the calibration object with identifier *2209ZW925B-4 (DA3mm)*.

1 Type of calibration

The short-circuit current I_{sc}, the open-circuit voltage V_{oc}, the fill-factor FF, the maximum power P_{MPP} and the efficiency η are determined as described in the IEC 60904-1:2020 standards. For this purpose the current-voltage characteristics of the calibration object is measured under standard test conditions as described in the IEC 60904-3:2019 standard. The irradiated area is defined using a mask.
The measurements performed on the calibration object and consequently also the measurement results are non-compliant to the application area specified in section 1 of the IEC 60904-1:2020 standard, since the present calibration object is a bifacial solar cell.

2 Calibration object

The calibration object with identifier *2209ZW925B-4 (DA3mm)* (see Fig. 1) is a solar cell with electrical contacts on both sides. The solar cell has a contact grid on the front side with 9 busbars. The rear side of the solar cell (see Fig. 1b) is partly metallized. The measurement is carried out using an aperture mask (ISFH identifier FN027) shadowing about 3 mm of the cell edges.
The calibration object is measured with the front side (FS) facing upwards.

Condition of calibration object

The visual examination of the calibration object did not detect any visible damage.

3 Measurement procedure

3.1 Spectral mismatch correction

For the determination of the spectral correction factor *MM* three measurement curves are required:

- The spectral responsivity of the calibration object. This measurement is traceable to the spectral responsivity of a primary calibrated WPVS reference solar cell (primary standard "DSR", see section 9).
- The spectral responsivity of the primary calibrated WPVS reference solar cell of the IV tester (primary standard "IV", see section 9).
- The spectral irradiance of the IV tester which is determined using a spectroradiometer in the wavelength range from 250 nm to 1700 nm.

The factor *MM* is calculated with these data as described in the IEC 60904-7:2019 standard using the reference spectrum of the IEC 60904-3:2019 standard.

3.2 Irradiance

The irradiance of the sun simulator is adjusted using a WPVS reference cell. The short circuit current of this reference cell was calibrated (primary standard "IV", see section 9) under standard test conditions (1000 W/m^2, AM1.5G reference spectrum and 25 °C).
For the adjustment of the irradiance the spectral correction factor *MM* is considered as defined in the IEC 60904-7:2019 standard (also see section 3.1).

3.3 Temperature of the calibration object and Voc-method

As defined in the IEC 60904-3:2019 standard the measurement will be carried out at a temperature of 25 °C. To achieve this temperature the Voc-method as described in Ref. 1 is used. The calibration object is mounted and contacted on the measurement chuck (see section 5). In the dark the temperature of the measurement chuck is adjusted until achieving $T_{\text{sample}} = 25.0\,°\text{C}$ stable in time. Afterwards the shutter of the lamp is opened and the time dependence of the open circuit voltage is monitored. The highest V_{oc} value from these data corresponds to the equivalent open circuit voltage $V_{\text{oc.equiv}}$ of the calibration object under 25 °C. Under illumination of the calibration object and a continuous measurement of the open circuit voltage the temperature of the measurement chuck is adjusted until achieving $V_{\text{oc.equiv}}$.

3.4 Correction of shadowing due to contacting bars

To correct for the shadowing of the contacting bars (see section 5) an equivalent short circuit current $I_{\text{sc.equiv}}$ is determined using a varying number of bars. For each combination of bars the short circuit current is determined. The equivalent short circuit current $I_{\text{sc.equiv}}$ follows by an extrapolation of the short circuit currents to zero contacting bars. Afterwards, the calibration object is contacted with all contacting bars and the short circuit current is measured continuously. The irradiance of the sun simulator is increased until the measured short circuit current equals $I_{\text{sc.equiv}}$.

3.5 Current-voltage characteristics

The determination of the current-voltage characteristics is carried out with a four-quadrant current-voltage source. A hysteresis between forward (from $V = 0$ to $V = V_{\text{oc}}$) and reverse (from $V = V_{\text{oc}}$ to $V = 0$) measurements was not observed, meaning that the relative deviation of the fill factor is smaller than 0.5 %.

One current-voltage characteristics consists of 165 voltage steps. Each step takes a time of 300 ms. Thus, the total acquisition time for the whole characteristics is 49.5 s. Please note that the data density is higher at and around $V = 0$, $V = V_{\mathrm{MPP}}$, $V = V_{\mathrm{oc}}$ and at the very steep part of the current-voltage characteristics between $V = V_{\mathrm{MPP}}$ and $V = V_{\mathrm{oc}}$. As a consequence, the voltage change from step to step is not constant over the whole characteristics.

4 Mounting

The calibration object is attached to a thermostatic chuck using a vacuum pump. The measurement chuck is adjusted such that the front surface of the calibration object is perpendicular and centrical to the optical axis. The measurement chuck is highly reflective.

5 Electrical contacting

The electrical contacting is realized using four-wire technique which uses separate current and sense leads.

5.1 Rear side

The gold-coated brass chuck, which is used to mount the solar cell, serves as electrical contact to the rear side. The gold-coated brass chuck is highly conductive and contacts the whole contact area of the solar cell's rear side.
Voltage-sensing is carried out using two rectangular parts (166 mm × 1.5 mm) of the chuck which are electrically isolated from the rest of the chuck.
This contacting-scheme neglects the resistance of the grid at the rear side.

5.2 Front side

The electrical contacting of the front side is carried out using contact bars which provide a continuous and elastic contacting area. By lowering the bars the contact area is pressed onto the busbar and establishes an electrical contact over the full length of the busbar.
For voltage measurement two small regions of the contact area at one of the center bars are isolated from the current-carrying part. Thus, voltage-sensing is carried out directly on the busbar. The position of the sense areas are at about one-fourth and three-fourth of the cell length.
This contacting-scheme neglects the resistance of the busbars.

6 Irradiance

6.1 Classification

The IV tester is classified as A+AA+ (spectrum, inhomogeneity of the light field and instability in time) according to the IEC 60904-9:2020 standard. This classification in all three areas is tested on a regular basis:

- The spectrum is tested before each measurement of the calibration object using a spectroradiometer. The spectroradiometer is traceable to reference lamps which were calibrated at the Physikalisch-Technische Bundesanstalt PTB (Braunschweig).
- The inhomogeneity of the light field is measured on a regular basis by scanning the light field with a reference solar cell.
- The instability in time of the light field is tested on a regular basis using a monitor diode.

The divergence of the light is smaller than 3°. The stability of the irradiance over time is controlled with a monitor diode during the whole measurement.

6.2 Irradiated area

The irradiated area is determined by a mask. The mask is realized by black cardboard and shadows about 3 mm of the cell edges. It was checked prior measurement that the cardboard fully absorbs the impinging light. The area of the aperture $A_{\text{aperture}} = (226.47 \pm 0.91)\,\text{cm}^2$ is measured using an optical scanning system working in transmission mode and is traceable to a primary calibrated area standard (primary standard “AREA”, see section 9).

7 Temperature measurement of the calibration object

The temperature T_{sample} of the calibration object is measured using a calibrated PT1000 temperature sensor which is integrated within the brass-chuck in a thinned area.

8 Measurement conditions

The temperature of the ambient during measurement is

$$T_{\text{room}} = (26.5 \pm 1.3)\,°\text{C}. \quad (1)$$

The relative humidity of the ambient during measurement is

$$H_{\text{room}} = (37.5 \pm 3.8)\,\%. \quad (2)$$

9 Used primary standards

The following area standards and WPVS reference solar cells, calibrated at the national metrology institute Physikalisch-Technische Bundesanstalt PTB (Braunschweig, Germany), were used as primary standards:

AREA: Calibration mark 50716-PTB-19 (FN001),

DSR: Calibration mark 47039-PTB-22 (RD007),

IV: Calibration mark 47086-PTB-21 (RD001).

10 Remarks

- The ISFH additionally provides the data in machine-readable format for further digital processing. However, only the data printed within this document are valid. It is up to the customer to carefully examine the electronic data before using them.
- Throughout the document a dot is used as decimal separator.
- The stability in time of the determined values was not tested.

11 Measurement result

The following results are obtained:

Short-circuit current I_{sc}:	(9178 ± 120) mA
Open-circuit voltage V_{oc}:	(747.1 ± 3.1) mV
Fill factor FF:	(85.33 ± 0.94) %
Maximum power P_{MPP}:	(5850 ± 99) mW
Efficiency η:	(25.83 ± 0.44) %

The uncertainty stated is the expanded uncertainty obtained by multiplying the standard uncertainty by the coverage faktor $k = 2$. The uncertainty analysis was carried out as described in the document EA-4/02 M:2013. The value of the measurand lies with a probability of 95% within the assigned interval.

12 Additional information

The following parameters are provided as additional information and thus do not necessarily include information about the measurement uncertainty.

Designated illumination area of cell $A_{designated}$:	(226.47 ± 0.91) cm^2
Spectral mismatch factor MM:	1.0019
Current at maximum power point I_{MPP}:	8809.4 mA
Voltage at maximum power point V_{MPP}:	664.1 mV

Figure 2 shows the measured IV characteristics under standard test conditions. The corresponding measurement data are provided in a machine-readable format. Due to the large number of data values the data are not printed within this certificate.

References

[1] K Emery, J Burdick, Y Caiyem, D Dunlavy, H Field, B Kroposki, T Moriarty, L Ottoson, S Rummel, T Strand, et al. Temperature dependence of photovoltaic cells, modules and systems. In *Photovoltaic Specialists Conference, 1996., Conference Record of the Twenty Fifth IEEE*, pages 1275–1278. IEEE, 1996.

003027
D-K-<br>18657-01-00
2022-11

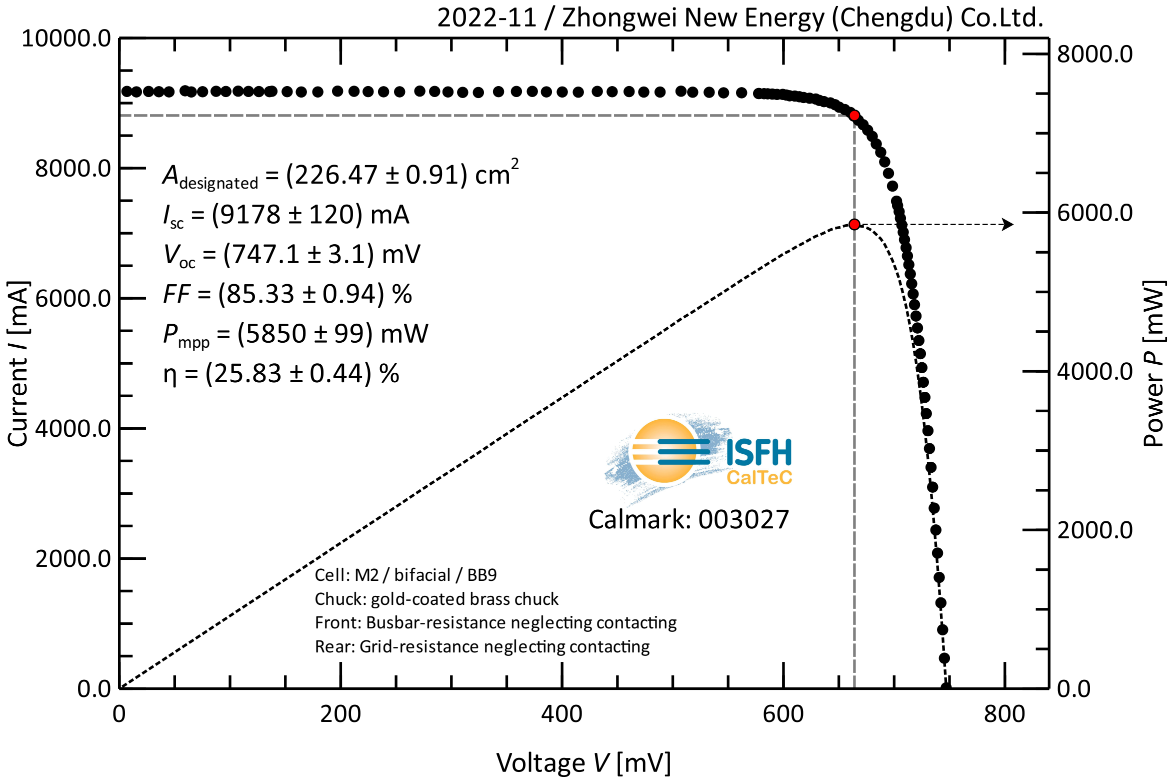

Fig. 2: Plot of the measured current-voltage characteristics under standard test conditions.

End of calibration certificate
