# Supplementary material for: Flexible solar cells based on foldable silicon wafers with blunted edges
Source: Nature. 2023 May 24;617(7962):717–23. doi: 10.1038/s41586-023-05921-z (PMC10208971; doi:10.1038/s41586-023-05921-z)

中国计量科学研究院
National Institute of Metrology, China

中国认可
国际互认
校准
CALIBRATION
CNAS L0502

校 准 证 书
Calibration Certificate

证书编号 GXgf2022-01614
Certificate No.

客户名称<br>Client	中国科学院上海微系统与信息技术研究所<br>Shanghai Institute of Microsystem & Information Technology Chinese Academy of Sciences (SIMIT)
器具名称<br>Instrument	太阳电池片<br>Solar cell
型号/规格<br>Type/Model	异质结 SHJ-M2-9BB-双面超薄电池<br>SHJ-M2-9BB solar cell
出厂编号<br>Serial No.	1#
生产厂商<br>Manufacturer	/
联络信息<br>Contact Information	上海市嘉定区城北路 235 号<br>235 Chengbei Rd, Shanghai, China
校准日期<br>Date of Calibration	2022 年 06 月 14 日
接收日期<br>Date of Receiving	2022 年 06 月 14 日

批准人：

Approved by

中国计量科学研究院 校准专用章

发布日期： 2022 年 07 月 29 日
Date of Issue

地址：中国北京北三环东路 18 号
Address：No.18 Bei San Huan Dong Lu,Beijing,P.R.China

邮编：100029
Post Code

电话：+86-10-64525569/74
Tel

传真：+86-10-64271948
Fax

网址：http://www.nim.ac.cn
Website

电子邮箱：kehufuwu@nim.ac.cn
Email

2019-jz-R0520

证书编号 GXgf2022-01614
Certificate No.

中国计量科学研究院（NIM）是国家最高的计量科学研究中心和国家级法定计量技术机构。1999年授权签署了国际计量委员会（CIPM）《国家计量基(标)准和国家计量院签发的校准与测量证书互认协议》（CIPM MRA）。The National Institute of Metrology (NIM) is China's national metrology institute (NMI) and a state-level legal metrology institute. NIM is China's signatory to the Mutual Recognition of National Measurement Standards and of Calibration and Measurement Certificates Issued by National Metrology Institutes (CIPM MRA) which is arranged by the International Committee of Weights and Measures (CIPM).

质量管理体系符合ISO/IEC17025标准，通过中国合格评定国家认可委员会（CNAS）和亚太计量规划组织（APMP）联合评审的校准和测量能力（CMCs）在国际计量局（BIPM）关键比对数据库中公布。NIM's quality management system meets requirements of the ISO/IEC 17025. Its Calibration and Measurement Capabilities (CMCs) that are peer reviewed both by China National Accreditation Service for Conformity Assessment (CNAS) and the Asia Pacific Metrology Programme (APMP) are published in the International Bureau of Weights and Measures (BIPM) Key Comparison Database (KCDB).

2020年，NIM和CNAS就认可领域的技术评价活动签署了谅解备忘录，承认NIM的计量支撑作用和出具的校准/检测结果的溯源效力。NIM and CNAS signed a Memorandum of Understanding (MOU) for Recognition of Technical Assessment in Laboratory Accreditation Field in 2020, in which CNAS recognizing the technical supporting role of NIM in laboratory accreditation and the traceability of NIM's calibration / test results.

校准结果不确定度的评估和表述均符合 JJF1059 系列标准的要求。The evaluation and expression of uncertainty of the calibration results are in line with the requirements of JJF1059 series standards.

校准所依据/参照的技术文件（代号、名称）Reference documents (Code,Name)

参照太阳电池校准规范：光电性能（JJF1622-2017）
According to JJF 1622-2017 Calibration Specification for Solar Cells: Photoelectric Properties

校准环境条件及地点 Calibration place and environment

温度 Temperature：	（24±2）℃	地点 Location：	和-13-110
湿度 Humidity：	（50±5）% RH	其它 Others：	

校准使用的计量基（标）准装置(含标准物质)/主要仪器
Reference Standards（Including the Reference Material）/ Instruments used

名称 Name	测量范围 Measurement Range	不确定度/准确度等级 Uncertainty/Accuracy	证书编号 Certificate No.	证书有效期至 Due Date (YYYY-MM-DD)
太阳能电池 IV 测试仪 IV tester	DC-V：(0-1)V<br>DC-I：(3-20)A	0.2%(k=2)<br>0.2%(k=2)	GXgf2021-13978	2022-11-30

2019-jz-R0520

证书编号 GXgf2022-01614
Certificate No.

名称<br>Name	测量范围<br>Measurement Range	不确定度/<br>准确度等级<br>Uncertainty/Accuracy	证书编号<br>Certificate No.	证书有效期至<br>Due Date<br>(YYYY-MM-DD)
稳态太阳模拟器<br>Steady-state solar simulator	(200-1500)W/m^2	AAA 级	GXgf2022-00197	2023-01-25
标准太阳电池<br>Reference solar cell	I_{sc}:100μA~200mA	0.9%(k=2)	GXgf2021-11879	2022-07-07
数字源表<br>Sourcemeter	DC-V：<br>10mV~10V<br><br>DC-I:100μA-1A	U_{rel}=(5×10^{-4}~<br>10×10^{-5})<br>(k=2)<br>U_{rel}=(10×10^{-5}~<br>40×10^{-5})<br>(k=2)	DCsy2021-12985	2022-12-30

证书编号 GXgf2022-01614
Certificate No.

校 准 结 果
Calibration Results

测量方法：
Calibration method：

利用稳态太阳模拟器（光谱失配度小于 5%），通过补偿探针遮挡对辐照度的影响，使得太阳电池片接收到的等效辐照度为 1000W/m^2，并控制电池片温度 25℃(通过半导体制冷台制冷，利用红外测温探头监控温度)，将稳态太阳模拟器快门打开，进行测量。电池片的性能参数测试结果如下：

Using a steady-state solar simulator (spectral mismatch less than 5%),by compensating the influence of the probe on the irradiance.The equivalent irradiance received by the solar cell is 1000W/m^2, control the solar cell temperature at 25 ℃ (by Infrared temperature probe). Open the shutter of the steady-state solar simulator for measurement. The key photoelectric parameters of the measured solar cell are as follows:

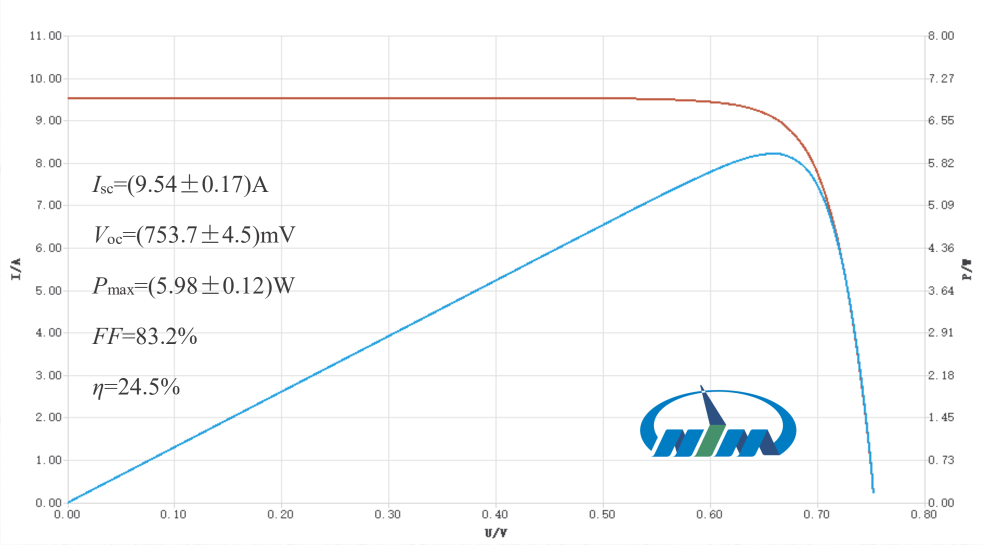

短路电流 I_{sc} (A)	开路电压 V_{oc} (mV)	最佳工作电流 I_{max} (A)	最佳工作电压 V_{max} (mV)	最大功率 P_{max} (W)	填充因子 FF (%)	电池片效率 η (%)
9.54	753.7	9.09	654.0	5.98	83.2	24.5

证书编号 GXgf2022-01614
Certificate No.

校　准　结　果
Calibration Results

不确定度描述:
Description of calibration uncertainty:
I_{sc}: 1.8% (k=2)；V_{oc}: 0.6% (k=2)；P_{max}:2.0% (k=2)

注：最佳工作电流、最佳工作电压、填充因子、电池片效率不作为量传参数。
Note: I_{max},V_{max},FF and η are not regarded as the parameters for value transfer.

计算电池片效率用的电池片面积由客户提供，为 244.3 cm^2。
The area of the solar cell used to calculate the efficiency is provided by the customer,which is 244.3 cm^2.

----------------------以下空白--------------------
Blank below

声明 Statement：
1.我院仅对加盖“中国计量科学研究院校准专用章”的完整证书负责。
NIM is ONLY responsible for the complete certificate with the calibration stamp of NIM.
2.本证书的校准结果仅对所校准的计量器具有效。
The certificate is ONLY valid for the calibrated instrument.
3.本证书用中英文两种语言表达，准确含义以中文为准。
The certificate is reported in both English and Chinese, with the Chinese version as standard.

校 准 员： 王[illegible]
Calibrated by

核 验 员： [illegible]
Checked by

Appendix: Summary of Certificate

NIM Certificate No.: GXgf2022-01614
Client: Shanghai Institute of Microsystem & Information Technology Chinese Academy of Sciences (SIMIT)
Sample: Solar cell
Type/Model:SHJ-M2-9BB solar cell
DUT S/N: 1#
Manufacturer:/
Date of Test: 06/14/2022
Temperature Sensor/Control System: None
Environmental conditions: (24±2) °C, RH (50±5) %

The calibration has been conducted by the PV Metrology Lab of NIM (National Institute of Metrology, China). Measurement of irradiance intensity and all other measurements are traceable to the International System of Units (SI). The performance parameters reported in this certificate apply only at the time of the test for the sample.

Area (cm^2)	I_{sc}(A)	V_{oc}(mV)	P_{max}(W)
244.3	9.54	753.7	5.98
I_{max}(A)	V_{max}(mV)	FF (%)	η (%)
9.09	654.0	83.2	24.5

I-V Characterization Methods:
JJF 1622-2017: Calibration Specification of Solar Cells：Photoelectric Properties
Secondary Reference Cell:
Device Material: Mono-Si
Solar Simulator:
Classification: AAA (Double-light source: Xeon and Halogen);
Total irradiance: 1000 W/m^2 based on I_{sc} of the above Secondary Reference Cell.

Note:The area of the solar cell used to calculate the efficiency is provided by the customer,which is 244.3 cm^2.

NIM Certificate No.: GXgf2022-01614

DUT S/N：1#

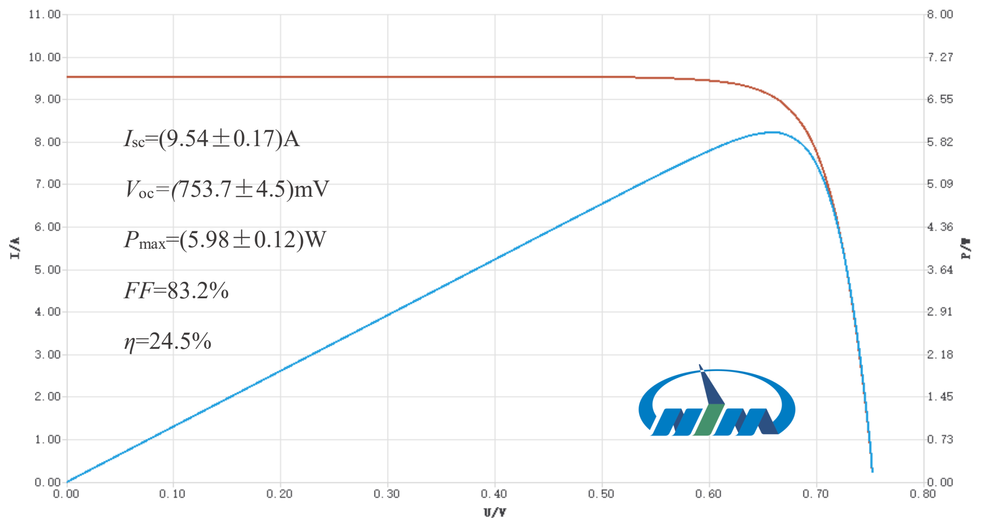

Supplement: Supplementary file 3 — This zipped folder contains Certificate Reports 1–3 and Vibrational Test Report. Descriptions of the four reports are also provided. [file 41586_2023_5921_MOESM3_ESM.zip › Supplementary Reports/Certificate report 1.pdf]
